# Assessing Systems Properties of Yeast Mitochondria through an Interaction Map of the Organelle

**DOI:** 10.1371/journal.pgen.0020170

**Published:** 2006-10-20

**Authors:** Fabiana Perocchi, Lars J Jensen, Julien Gagneur, Uwe Ahting, Christian von Mering, Peer Bork, Holger Prokisch, Lars M Steinmetz

**Affiliations:** 1 European Molecular Biology Laboratory, Heidelberg, Germany; 2 Institute of Human Genetics, Technical University, Munich, and GSF National Research Center for Environment and Health, Neuherberg, Germany; Stanford University School of Medicine, United States of America

## Abstract

Mitochondria carry out specialized functions; compartmentalized, yet integrated into the metabolic and signaling processes of the cell. Although many mitochondrial proteins have been identified, understanding their functional interrelationships has been a challenge. Here we construct a comprehensive network of the mitochondrial system. We integrated genome-wide datasets to generate an accurate and inclusive mitochondrial parts list. Together with benchmarked measures of protein interactions, a network of mitochondria was constructed in their cellular context, including extra-mitochondrial proteins. This network also integrates data from different organisms to expand the known mitochondrial biology beyond the information in the existing databases. Our network brings together annotated and predicted functions into a single framework. This enabled, for the entire system, a survey of mutant phenotypes, gene regulation, evolution, and disease susceptibility. Furthermore, we experimentally validated the localization of several candidate proteins and derived novel functional contexts for hundreds of uncharacterized proteins. Our network thus advances the understanding of the mitochondrial system in yeast and identifies properties of genes underlying human mitochondrial disorders.

## Synopsis

Mitochondria are organelles which are best known as the cell's energy powerhouses. They have a special evolutionary origin derived from bacteria engulfed about 2 billion years ago by eukaryotes. Surprisingly, mitochondrial functions have been retained over evolution, so that unicellular yeast and multicellular organisms like humans share many of the same mitochondrial components. Here the authors complemented previous efforts to identify the “parts” of the mitochondrial system, but as for any system, this is not enough to understand how it works. By integrating information on protein localization, function, and interaction, the authors go a step further and propose a map of the mitochondrial organelle and its surroundings. This map suggests the involvement of hundreds of so far uncharacterized proteins in mitochondrial function. By taking advantage of the high conservation of the organelle to humans, the authors investigate properties of human genes involved in mitochondrial diseases. They find that the disease genes have ancient origin and a mild mutant phenotype when their function is abolished in yeast. The approach applied here can be extended to other organelles or organisms and illustrates a growing trend in understanding biological processes in their whole rather than in isolated parts.

## Introduction

Mitochondria play a central role in metabolism, energy production, ion homeostasis, and apoptosis [1,2] and are found in most eukaryotes. Not surprisingly, a large fraction of all characterized human Mendelian disease genes encode proteins localized to mitochondria [3]. It is estimated that 700–800 mitochondrial proteins are present in Saccharomyces cerevisiae with a higher number in humans [2,4]. It is widely accepted that mitochondria originated from an endosymbiosis between an ancestral alpha-proteobacterium and a eukaryotic host [5,6]. During evolution most of the genes encoded by the mitochondrial genome (mtDNA) were transferred to the nucleus or were lost [7]; now only eight proteins are encoded by the mtDNA of yeast and 13 in humans. Thus, despite having their own genome, mitochondria are highly dependent on extra-mitochondrial processes for their function and biogenesis.

Genome-scale approaches have catalyzed the identification of mitochondrial proteins in different organisms through, for example, analysis of deletion phenotypes [8,9], subcellular localization [10–12], gene expression [4,13–15], and mass spectrometry-based proteomics [4,16–21]. Each systematic dataset surveyed different properties of mitochondrial proteins, identifying proteins physically residing in mitochondria and genes functionally related to the organelle. A comparison of two datasets in mouse [18] and 22 datasets in yeast [4] demonstrated that indeed different sources of experimental evidence clearly provide diverse degrees of complementary information on mitochondrial localization, phenotype, and regulation. In fact the integration of all data types can overcome the limited sensitivity and specificity of each dataset individually and can result in a more accurate catalog of mitochondrial associated proteins [4]. Similar integrated analyses have been applied recently to identify human mitochondrial proteins, despite a much smaller collection of available genome-wide datasets [22,23].

Nevertheless, characterization of the mitochondrial proteome is still incomplete. To date, 533 proteins are verified as localized to the mitochondrial organelle in yeast by single-gene studies [22]. Yet even with a set of 533 annotated mitochondrial proteins, about one third of the expected 800 proteins still remains to be verified. Beyond mitochondrial protein identification, the functional role of all proposed candidates remains to be explored in the context of known mitochondrial proteins.

Protein networks, which describe the interrelationships among components, provide a context to functionally characterize candidate proteins. Functional links between proteins have been defined based on physical interactions [24–29], expression regulation [30–33], mutant phenotypes [8,34], phylogenetic profiles [35], literature mining [36], and orthology transfer of interaction evidence across species [37,38]. Analogous to the identification of mitochondrial proteins, integrating heterogeneous but complementary interaction data types improves the accuracy and the coverage in detecting protein associations [39] and has been implemented globally [40–46]. However, a comprehensive network reconstruction for mitochondria is missing and moving from a list of proteins to their placement into a functional context is needed.

Here, we analyze the yeast mitochondria at a systems level, first by defining an accurate and comprehensive list of mitochondrial components and then integrating it with diverse data sources on protein associations to construct a network of functional interactions. The network yields a comprehensive map of mitochondrial modules and a functional context for hundreds of uncharacterized components. Analyses of systems properties—conditioned by, but not easily deduced from the individual parts of the system—reveal hypotheses about expression regulation and evolution. Our survey has implications beyond yeast, for candidate gene identification of human mitochondrial disorders.

## Results/Discussion

**Figure 1:**
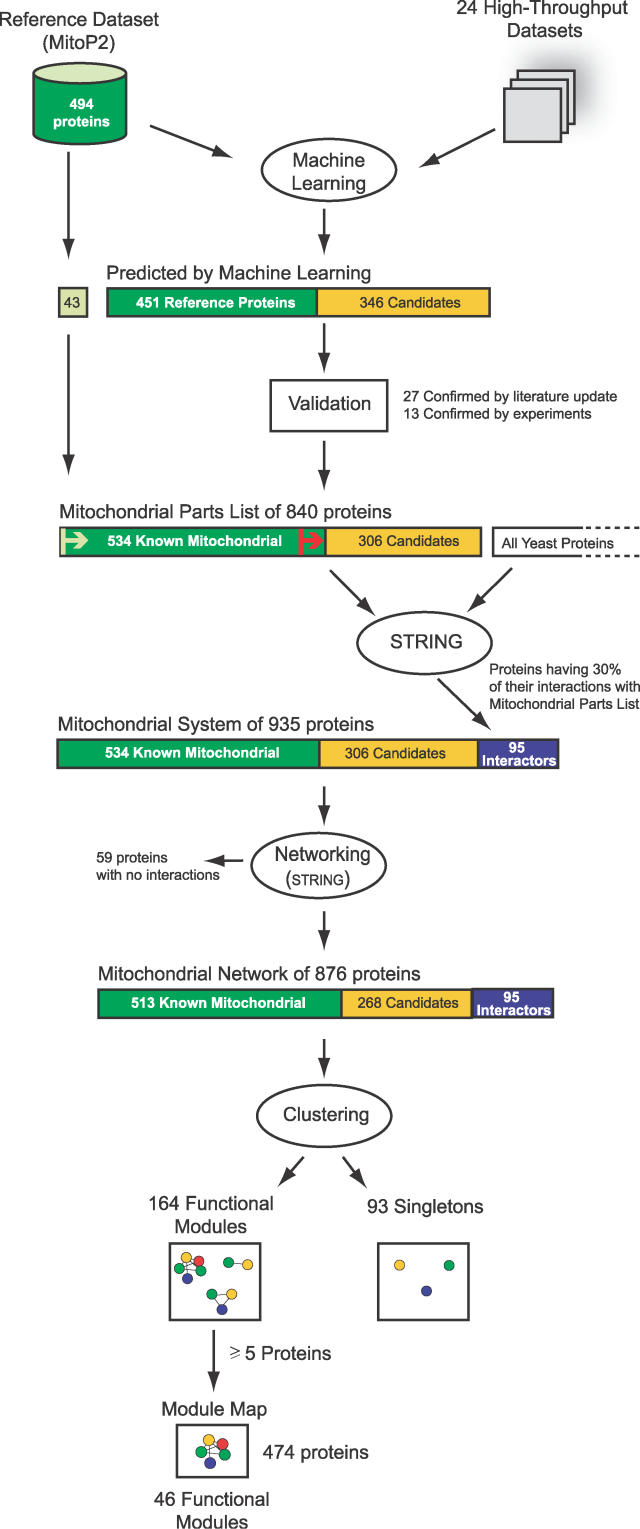
Summary of the Integrated Approach

Our approach to predict a mitochondrial parts list, derive a functional network of yeast mitochondria, and identify mitochondrial functional modules is depicted in Figure 1.

### Mitochondrial Parts List

To predict a high-quality set of mitochondrial proteins, we collected 24 genome-wide datasets that represent complementary approaches to study the mitochondrial organelle (Table S1). Of these datasets, 22 are the same as in our previous study where their sensitivity and specificity were assessed [4]. Here, to integrate all datasets, we applied a machine learning approach where a linear classifier was trained to discriminate between 494 known mitochondrial localized proteins (reference set) and the remaining set of yeast proteins (see Materials and Methods). The linear classifier computes a score for each input dataset (Table S1), which can be used to rank the datasets according to their power in predicting a mitochondrial localization. Sub-cellular localization by protein-tagging [10,11], protein identification by mass spectrometry of mitochondria [4,16], deletion phenotype screening [8,9], expression profiling of a transcription factor mutant [15], and orthology to species-specific collections of known mitochondrial proteins (humans, Neurospora crassa) were the most informative data sources, together with orthology to *Rickettsia prowazekii* and the absence of orthology to *Encephalitozoon cuniculi,* a eukaryotic parasite that lacks mitochondria. In contrast, transcriptome analysis of respiratory versus fermentative conditions [4,13], low confidence protein interactions with known mitochondrial proteins [39], and analysis of transcripts associated with mitochondrion-bound polysomes [14] were not informative predictors of mitochondrial localization.

The linear classifier was then used to integrate the datasets and to compute a composite score of mitochondrial localization for every gene product in the yeast genome (Table S1). We compared the predictions from our machine learning method to two published methods [4,22], as well as to annotations for mitochondrial proteins in the Saccharomyces Genome Database (SGD) (http://www.yeastgenome.org) (see Materials and Methods and Figure S1). Our machine learning approach performed substantially better than the original MitoP2 predictions [4], even when the latter algorithm was applied to our 24 datasets; it also performed better than the SGD annotations and at least as well as the more recent MitoP2 predictions by a support vector machine approach [22] (see Figure S1).

By analyzing the sensitivity of the linear classifier in recalling proteins in the reference set at multiple score thresholds, we selected the top 800 scoring proteins (score higher than 0.413, see Text S1). This threshold was chosen because a decrease in prediction performance occurs after this point (see Figure S1), it properly predicts nearly all (91%) of the reference set proteins, and the total number is close to the estimated size of the mitochondrial proteome [2,4]. Among the few proteins (9%) in the reference set that were not captured are intron-derived gene products from the mitochondrial genome and dual localized proteins like Pop1p, Pop3p, Pop4p, Pop5p, Pop6p, Pop7p, Pop8p, and Rpp1p of the ribonuclease mitochondrial RNA-processing complex (RNase MRP) [47]. In addition to the known mitochondrial proteins, the top 800 scoring proteins included an additional 346 proteins not in the reference set (three proteins annotated in the SGD database as hypothetical and derived from dubious open reading frames were omitted, see Table S1).

**Figure 2:**
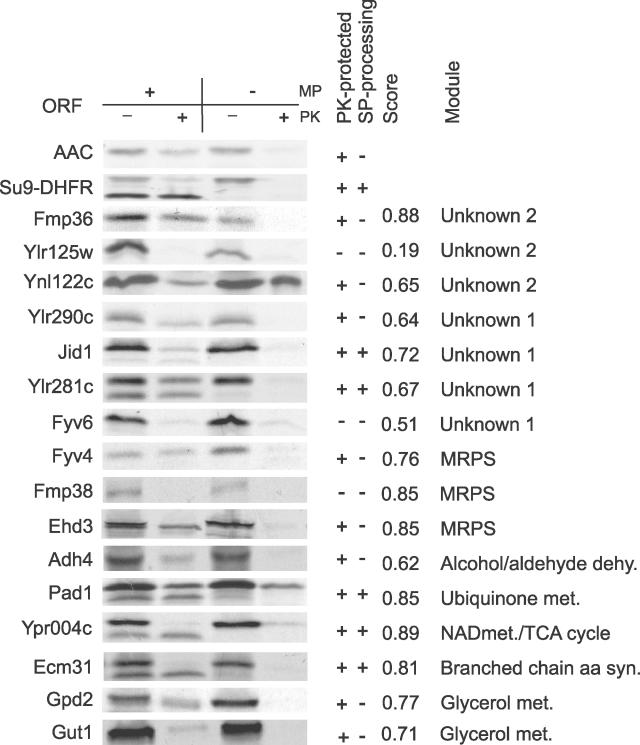
Verification of Predicted Mitochondrial Candidates by Mitochondrial Import Samples were derived by incubating radiolabeled proteins with isolated mitochondria in the presence or absence of a membrane potential and of proteinase K. Cases where import was accompanied by removal of the signal peptide are marked as ‘‘SP-processing'' (+). Su9(1–69)DHFR and AAC serve as positive controls for a processed matrix protein and a non-processed inner membrane protein, respectively. The score reflects the likelihood of mitochondrial localization for tested candidates as predicted by the linear classifier. MP, membrane potential; PK, proteinase K; SP, signal peptide.

Our prediction of a mitochondrial localization for these 346 candidates is supported by several lines of evidence. First, 38 proteins have recently been annotated as mitochondrial localized in MitoP2 [22], of which 27 (75%) are predicted as candidates by the linear classifier. Second, our set of mitochondrial candidates shows remarkable overlap with a recent large-scale proteome analysis, which identified 851 yeast mitochondrial proteins using mass spectrometry [17]: 64% of our candidates (221 of 346) are also reported by this study, which notably was published after the prediction of our list. Third, we experimentally tested mitochondrial localization of 16 candidates, with a wide range of scores, by import into isolated mitochondria (Figure 2). In addition to the two known mitochondrial proteins that served as positive controls, 13 of the 16 candidates were imported into mitochondria, providing evidence to the high confidence of our prediction method.

The 346 candidates include a substantial number of previously uncharacterized proteins. Only 28% of these candidates (96/346) have high-confidence gene ontology (GO) annotations. While some link to known mitochondrial pathways, many others belong to processes that are not traditionally associated with the organelle; for example, proteins involved in vacuolar acidification, glycerol metabolism, and nuclear DNA metabolism (see below). Remarkably, even when considering all GO evidence types, nearly half of our candidates (146 of 346), are entirely uncharacterized.

### Mitochondrial Context

To define the extra mitochondrial context for our system, we used the Search Tool for the Retrieval of Interacting Genes/Proteins (STRING) (http://string.embl.de/version_6_2). Previously established criteria [48] were applied to identify reliable physical protein-protein interactions. By searching for proteins that have more than 30% of their high-confidence physical interactions to proteins of the mitochondrial parts list (see Materials and Methods) we identified 95 additional proteins. These proteins were added to the parts list to define our mitochondrial system, containing a total of 935 proteins.

### Mitochondrial Network and Functional Modules

**Figure 3:**
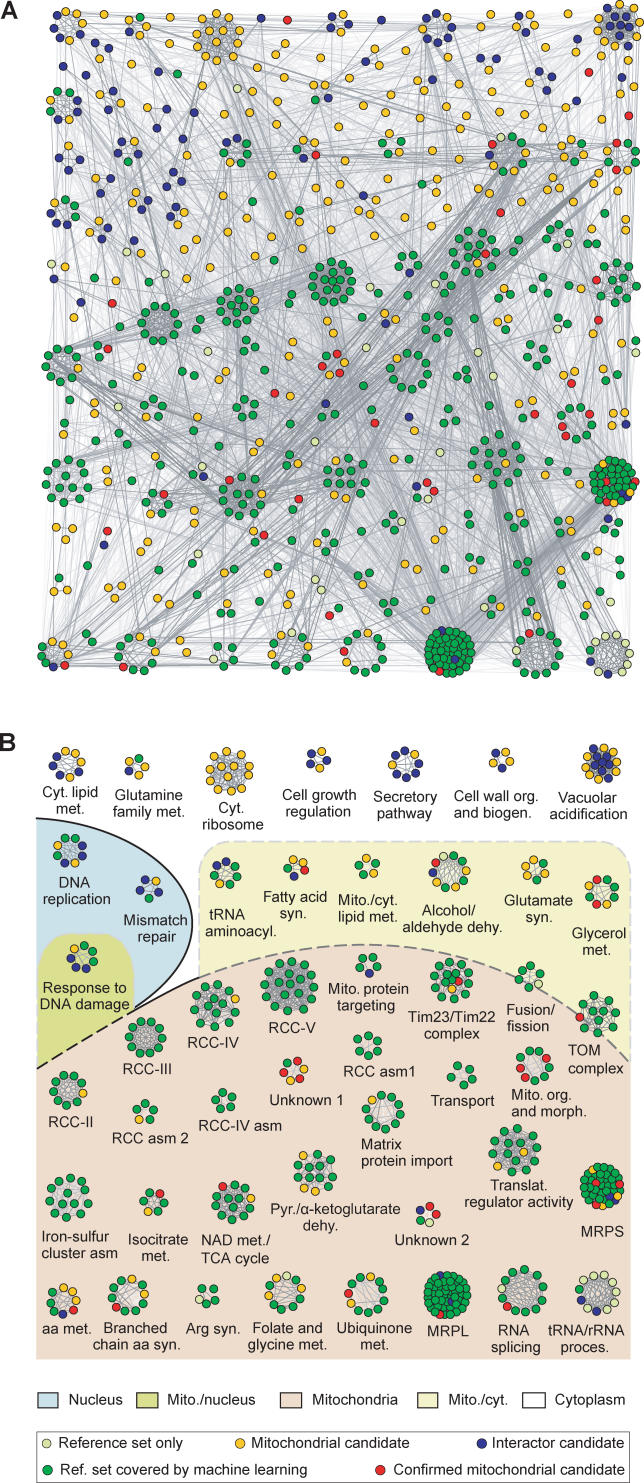
Functional Network of the Mitochondrial System (A) Full network containing 9,780 association lines connecting 876 protein nodes. Lines are shaded by the degree of STRING confidence in the association. Nodes are colored according to the following: known mitochondrial-localized proteins (reference set) correctly predicted by the linear classifier, green; known mitochondrial-localized proteins not captured by the linear classifier, light green; predicted proteins not annotated as mitochondrial-localized (mitochondrial candidates), orange; proteins predicted as additional interactors by the network (interactor candidates), blue; mitochondrial candidates recently annotated as mitochondrial-localized (MitoP2 database) or verified by mitochondrial import assay, red. (B) Module map of 46 modules with five or more proteins. Modules were named and localized based on GO terms, with the following abbreviations: asm, assembly; biogen, biogenesis; cyt, cytoplasmic; dehy, dehydrogenase; met, metabolism; mito, mitochondrial; org, organization; proces, processing; syn, synthesis. The localization of modules in three different compartments—nucleus, mitochondria, and cytoplasm—is indicated by sectors of different colors. When the module contains a mixture of proteins with different localization it is annotated as shared between the different compartments. Module shared between mitochondria and nucleus or mitochondria and cytoplasm belong to green and yellow sectors, respectively. Cytoplasm refers to all of the contents of a cell excluding mitochondrion and nucleus but including the plasma membrane and other sub-cellular structures. The identity of all proteins and their functional links can be found in Figure S4 and Figure S5 for (A and B), respectively, where the standard gene names are shown within the nodes and are hyperlinked to STRING.

To construct a global model of the yeast mitochondrial network, we combined our protein list with evidences of physical and functional interactions from diverse data sources reported in STRING (see Materials and Methods). STRING also provides confidence scoring of associations through a benchmarked integration of physical protein-protein interactions, pathway databases, literature mining, expression analysis, and genomic context methods, all transferred across about 200 organisms [38]. The integration resulted in a network that has 9,780 linkages and provides a functional context for 876 proteins, more than 90% of the mitochondrial system (Figure 3A): 513 of the 534 with confirmed mitochondrial localization, 268 of the 306 remaining mitochondrial candidates, and all of the 95 interactor candidates (see Table S2, Figures S3 and S4 for interaction evidence).

To obtain a map of the biological processes captured, we then performed hierarchical average-linkage clustering using the STRING confidence scores of the network associations as a similarity measure. After carefully evaluating different cutoff values for defining modules (see Materials and Methods), we set a similarity cutoff that yields 164 functionally distinct modules with two or more proteins (783 proteins) and 93 singletons.

These modules group proteins with similar functions (see GO terms associated with each module in Table S3); therefore, poorly characterized proteins within these modules may share a related function. More than 70% of mitochondrial candidates of unknown biological processes could be placed into a functional context through our network; thus providing the first clues to their function. For example, the hypothetical protein Ypl109cp likely plays a role in the biosynthesis of ubiquinone. This protein has an ortholog in Rickettsia prowazekii and has been shown to localize to mitochondria by high-throughput studies [11,16,17]. In several proteobacterial species, including alpha-proteobacteria, the gene is predicted with high confidence to be in an operon together with *COQ5,* which is known to be involved in ubiquinone metabolism. Moreover, Ypl109cp contains an ABC1 domain associated with ubiquinone biosynthesis. All of these reasons make Ypl109cp an attractive candidate to play a role in ubiquinone metabolism.

Of the 164 functional modules, 46 contained five or more proteins (altogether 474 proteins) and were analyzed further (Figure 3B and Figure S5; see Text S1 for a description of individual modules). The analysis of sub-cellular localization revealed that, as expected, the majority of these modules are found in mitochondria (30 of 46). Many of these modules are known mitochondrial complexes, such as the respiratory chain complexes; other modules include proteins involved in metabolic pathways, such as iron-sulfur cluster assembly and the TCA cycle. The remaining 16 modules represent processes related to mitochondrial function and biogenesis and are discussed below. This module map provides an advantage over a list of genes because it enables an overview of the entire system.

### Evaluation of Functional Modules

We expected our module map, which focuses on a specific sub-cellular system, to be more accurate than public databases that provide annotation at the genome level. In order to evaluate and estimate the level of agreement between our reconstructed functional network and public annotations we asked whether our modules are annotated in SGD, comparing to protein complexes, and in KEGG (Kyoto Encyclopedia of Genes and Genomes), comparing to metabolic pathways (see Materials and Methods). Using the criteria that 50% of the proteins in our modules are contained in an annotated protein complex or pathway, we could match 23 of the 46 modules to annotated pathways in KEGG and 13 to protein complexes in SGD. Good agreement was found for well-known mitochondrial processes; for example, complexes that define the oxidative phosphorylation chain, the mitochondrial membrane translocase machineries, and the TCA cycle. However, 17 functional modules were not matched by either of the two databases, including the iron-sulfur cluster assembly module, which contains proteins known to physically interact [49] and the fission and fusion module, which contains components known to genetically interact [50]. These unmatched modules point to limitations in both of the current databases.

In addition, our approach predicted two novel mitochondrial modules, Unknown 1 and Unknown 2, in which most of the proteins are uncharacterized. All of these proteins are grouped together primarily due to co-expression associations (Figure S3). While the evidence linking these proteins together is limited, the prediction for mitochondrial localization was strong. Therefore, we analyzed these modules by protein import experiments. Module Unknown 1 contains five mitochondrial candidates: Ylr281cp, Ylr283wp, Jid1p, Fyv6p, and Ylr290cp, and a known mitochondrial protein, Cox16p, involved in respiratory chain complex assembly. Protein import experiments confirmed a mitochondrial localization for three out of four tested candidates. Similarly, for module Unknown 2, even though the interactor candidate of this module, Ylr125wp, was not imported, we were able to confirm import of the two mitochondrial candidates (Figure 2).

### Mitochondrial Connectivity to the Cell

As mitochondria rely on cellular processes for their biogenesis and function, it is essential to characterize the organelle as an integrated unit in the cell rather than in isolation. To this effect, our network reveals two classes of mitochondria-cellular associations. First, it correctly includes several processes that take place outside the organelle that have an effect on mitochondrial function: it contains two modules whose proteins primarily localize to the nucleus and seven whose proteins primarily localize to the cytoplasm (respectively, blue and white sector in Figure 3B). One example is the DNA replication module, where the activity of the ribonucleotide-diphosphate reductase complex in the nucleus has been shown to affect mitochondrial DNA replication and repair through alterations of deoxyribonucleotide triphosphate pools [51].

Second, the network contains modules with components that are dual localized or components of enzymatic pathways that have branches inside and outside mitochondria. In this category, the network contains six modules that represent well-characterized enzymatic pathways common between the mitochondria and the cytoplasm: tRNA aminoacylation, fatty acid biosynthesis, lipid metabolism, alcohol and aldehyde dehydrogenases, glutamate biosynthesis, and glycerol metabolism (yellow section in Figure 3B). Several new hypotheses can be made from these modules. The glycerol metabolism module, for example, includes known cytosolic enzymes such as Gut1p, Gpd1p, and Gpd2p that oxidize glycerol-3-phosphate. Their activity and regulation is also important to create a glycerol-3-phosphate shuttle involved in redox adjustment during respiration [52]. Interestingly, although Gut1p and Gpd2p are known cytosolic enzymes, they are predicted mitochondrial candidates in our network. To test this prediction, we performed mitochondrial import experiments and confirmed import of both Gut1p and Gpd2p, implying a novel dual cytosolic and mitochondrial localization (Figure 2). These results support an intricate association of mitochondria with their cellular surroundings.

### Mutant Phenotypes of Functional Modules

**Figure 4:**
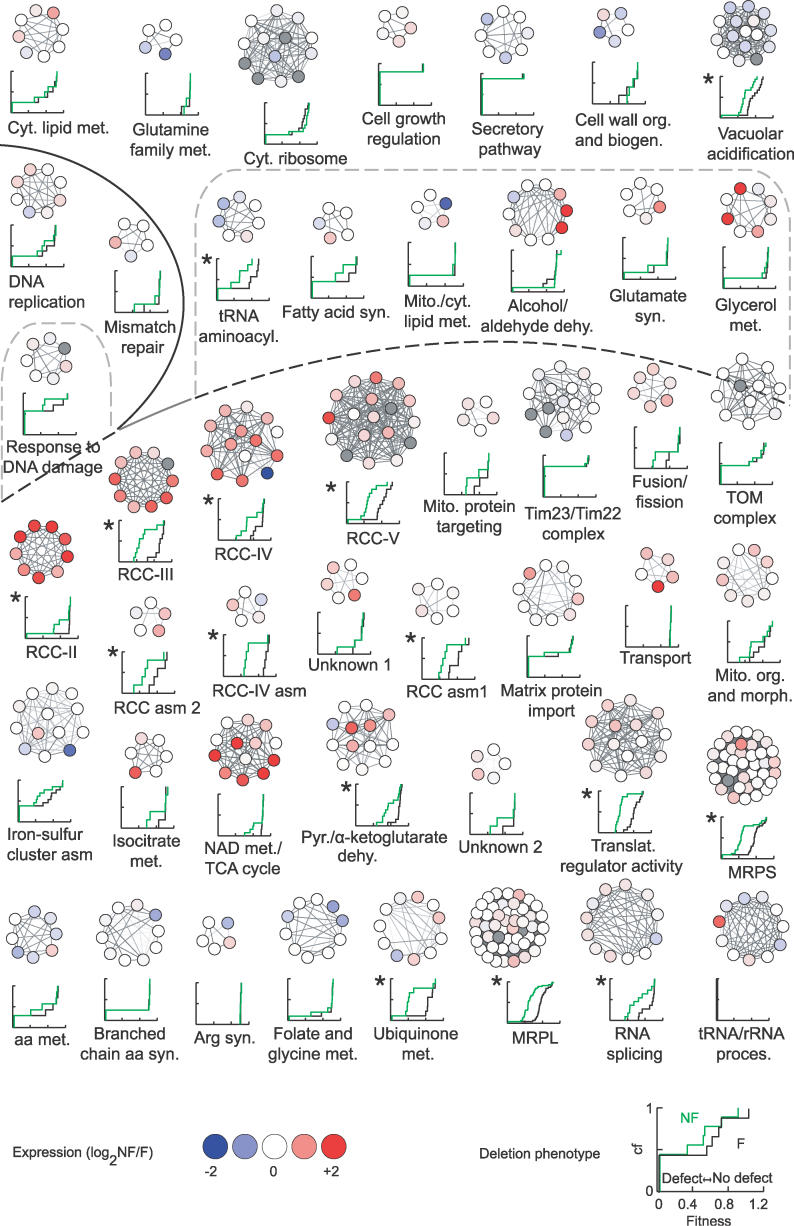
Mutant Phenotype and Regulation Cumulative frequency of the fitness scores of single gene deletion mutants on NF, green line, and F, black line, conditions are plotted for each functional module. Genes annotated in SGD as inviable were assigned a fitness value of zero. As an example, the graph depicted in the legend shows that close to half of the genes are essential (lower half of y-axis), the other half differ in their quantitative deletion phenotypes and range from poor fitness (severe growth defect) to high fitness (no growth defect). On the x-axis, the offset of the curves of different color shows that the distribution of growth defects of the non-essential genes differs between the two growth conditions, and in this case shows that more severe defects were observed under NF than F conditions. This designates a mitochondrial specific phenotype. The one-sided paired Wilcoxon rank test was used to assess whether the deletion mutant strains for genes of a module have a significantly impaired fitness under NF versus F conditions. Significance is indicated by star (*p* < 0.05). In the modules, node color indicates the mRNA expression level difference between growth on NF (YPL, SCL, and YPE) versus F (YPD and SCD) conditions (red gradient, higher expression; and blue gradient, reduced expression during NF growth). A stringent criterion was applied to calculate a single expression value for the difference between NF and F growth: genes with a consistent direction of the three ratios were assigned the least-fold difference; genes that show no differential regulation or show fold ratios opposite in direction between any two conditions were assigned a fold ratio of zero. Gray nodes had no expression measurements. The identity of single proteins can be found in Figure S6. cf, cumulative frequency.

To survey properties of the mitochondrial system, we further analyzed the network at the module level. We used a genome-wide dataset on the fitness of single-gene deletion mutants grown on non-fermentable (NF) and fermentable (F) substrates [8] to generate a phenotype profile for each module (Table S4). This dataset was not used to capture network associations. Mutants with respiratory defects have specific growth impairment on NF substrates, where mitochondrial respiration is required for optimal growth. From the quantitative deletion data we compared the fitness distributions under NF and F growth conditions for each module (Figure 4; see Figure S6 for gene names). These phenotype profiles provided two characteristics: the general essentiality of module components for cell viability and their specific involvement in respiration. A statistical test to assess the significance of the impaired fitness under NF versus F conditions for genes within the same module was performed.

Among modules localized within mitochondria, proteins in the metabolic modules seem dispensable for growth in both rich NF and F conditions. Most of the modules involved in mtDNA transcription and translation, respiration, and energy metabolism have significantly greater impaired fitness in NF than F carbon sources. Notably, mitochondrial modules associated with import and processing of proteins across the inner and outer mitochondrial membrane, and iron-sulfur cluster assembly are significantly impaired in both growth conditions. These modules represent functions of mitochondria essential for cell viability (Figure 4).

Not surprisingly, respiratory defects were the most frequent among the mitochondrial localized modules. Therefore, we tested if deletion strains of components in the two newly identified modules, Unknown 1 and Unknown 2, would also lead to an impairment of respiratory capacity. These components were grouped by co-expression evidence and show similar expression profiles to mitochondrial ribosomal proteins needed to translate mtDNA-encoded respiratory chain subunits. The measurements of O_2_ consumption of the deletion strains did not indicate any respiratory impairment. The same conclusion was reached when analyzing the biochemical activity of the respiratory chain complexes II and III and complex IV (unpublished data), arguing that the new modules might not be involved in energy metabolism or their components have a redundant function.

Furthermore, the deletion analysis implicates some extra-mitochondrial modules in respiration. For example, the vacuolar acidification module, which is important for many cellular processes including endocytosis, targeting of newly synthesized lysosomal enzymes, and the uptake of metal ions, has a specific deletion phenotype in NF media. Our genome-wide data on this module are supported by single-gene studies of yeast mutants that do not grow on NF media due to an increased sensitivity to iron [53,54]. Therefore, the phenotype profiles of modules reveal different degrees of involvement for functional processes in respiration.

### Expression Regulation

Our previous study [4] generated mRNA expression profiles under NF and F conditions that were here mapped onto the network to analyze the extent to which the mitochondrial system is regulated (Table S4). This dataset did not contribute to the parts list prediction as it was uninformative for that purpose (Table S1; see Text S1). Genome wide, 287 genes showed an expression change above 2-fold: 221 being elevated and 66 reduced in expression under NF conditions. Of these, 63 elevated and six reduced genes were present among the mitochondrial system (Figure 4).

Globally, poor correlation had been reported between gene expression and deletion phenotype [55,56]. However, when restricting the comparison to the set of proteins in the mitochondrial system, we observed a significant correlation between both approaches profiled under the same, NF and F, conditions (Spearman-rank test, *p* < 0.001; notably, this comparison does not suffer from circular reasoning because the expression dataset was uninformative in defining the parts list, see Materials and Methods). That is, genes that increased expression also showed a fitness defect upon gene deletion, more often than expected by chance.

Genes within modules often had different degrees of expression regulation. For example, the respiratory chain complex IV has two interchangeable subunits, *COX5a* and *COX5b,* which are oppositely regulated. The *COX5a* gene is expressed under aerobic conditions and *COX5b,* which supports a higher turnover rate, is up-regulated when cells experience hypoxia [57]. Similarly, the catalytic subunits of the respiratory chain complexes show higher regulation than subunits involved in their assembly or stability. This suggests that actually only a few subunits may be transcriptionally regulated in order to control the action of entire complexes, similar to what was proposed for complexes involved in the yeast cell cycle [48]. If such regulation occurs, it can be predicted that a substantial difference in deletion phenotype between NF and F conditions would occur for the regulated key components, which indeed was observed (same as above).

### Evolution, Conservation, and Disease Susceptibility

**Figure 5:**
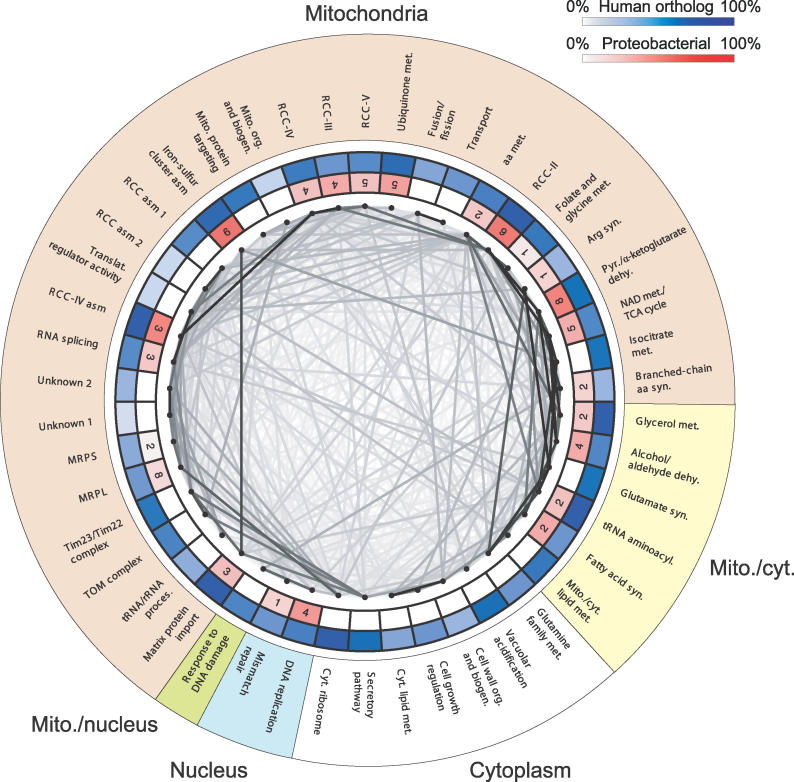
Origin and Conservation of the Mitochondrial System Orthology was used to identify functional processes in yeast that originate from bacteria and are conserved to humans. Modules of five or more proteins are shown as single nodes at the circumference of the circle and the degree of evidence connecting modules is shown by lines in different gray tone. The connection between modules is calculated as the average of the STRING interaction scores for all protein-pairs. Modules are distributed according to their localization. Color in the inner rings reflects for each module the percentage of proteins that have proteobacterial orthology (red) or human orthology (blue). The number of proteobacterial orthologs is also indicated.

Evolutionary conservation of mitochondria to proteobacteria was analyzed by using a set of orthologs inferred from a phylogenetic comparison of alpha-proteobacterial and eukaryotic genomes [58]. Proteobacterial ancestry was detected for 13% (119/935) of the proteins in the mitochondrial system. 23 modules contained at least one proteobacterial ortholog, of which 17 are localized to mitochondria and are related to respiratory metabolism (respiratory chain complexes, mitochondrial ribosome, RNA splicing, pyruvate metabolism, TCA cycle) as well as metabolism of amino acids and cofactors (Figure 5).

Analysis of conservation to humans yielded orthologs for 60% (565/935) of the proteins in the yeast mitochondrial system (Table S5), which is significantly higher than the conservation found for the entire yeast proteome (46%), even when corrected by the contribution of human orthology in the linear classifier (corrected *p*-value, hypergeometric test, *p* < 10^−15^, see Text S1). Overall, conserved proteins were distributed over most modules (Figure 5). Still, we detected lack of conservation for specific modules, which reflects functional differences between yeast and human mitochondria in the inheritance of the mitochondrial genome [59] and in the amino acids synthesized.

Human mitochondria have been predicted to house up to 2,000 different proteins [2], of which about half are annotated as mitochondrial reference set in the MitoP2 database, and about 150 have been implicated in human Mendelian disorders [22]. To investigate disease susceptibility, we identified network proteins that are orthologs to human disease genes (Swiss-Prot database) (Table S5). Of the 99 identified yeast proteins, 73 associate with an annotated Mendelian mitochondrial disorder (Table S4). The remaining 26 proteins associate with diseases for which a link to mitochondrial pathophysiology is plausible but not proved. These remaining proteins belong to modules involved in DNA replication and repair and biosynthesis of ubiquinone, folate, lipids, and amino acids.

**Figure 6:**
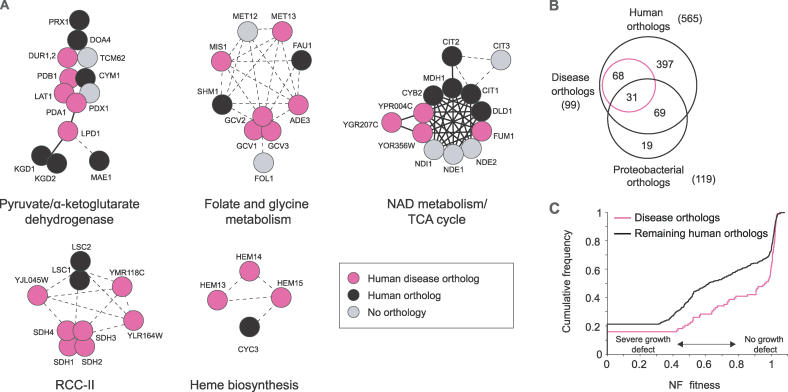
Properties of Yeast Orthologs to Human Disease Genes within the Mitochondrial System (A) Five modules enriched in human disease gene orthologs. Node color identifies human orthologs to yeast genes with and without associated Mendelian diseases (OMIM database). Proteins that belong to physical complexes are shown by overlapping nodes or in some cases are connected by solid lines; functional associations are shown by dotted lines. Disease genes within the same functional module had a tendency to have similar clinical phenotypes: glutaricaciduria II (NAD metabolism/tricarboxylic acid cycle), glycine encephalopathy (folate and glycine metabolism), mainly susceptibility to hereditary pheochromocytoma and paraganglioma (RCC-II), and variants of inherited disease of porphyrin metabolism (heme biosynthesis). Table S6 contains descriptions for each disease gene. (B) Conservation of disease genes to proteobacteria. Venn diagram of the overlap of yeast genes having human orthologs, proteobacterial orthologs, and human disease gene orthologs. Of the proteins with a disease ortholog, 31% (31/99) have a proteobacterial ortholog—whereas only 18% (100/565) of all human orthologs have a proteobacterial ortholog (hypergeometric test, *p* < 10^−4^). (C) Fitness distribution of yeast orthologs of disease genes. Empirical cumulative distributions of NF fitness scores are shown for all human disease gene orthologs compared to the remaining human orthologs. NF fitness represents the growth and survival of single gene deletion mutants under respiratory conditions (NF carbon source): higher fitness represents a milder growth defect. As a group, disease orthologs have higher fitness (i.e., milder mutant phenotypes) (*t*-test, *p* < 0.01).

Six modules had three or more distinct human disease orthologs, covering alone 29% of the disease gene set (28 of 98). Five of these modules localize to mitochondria and are involved in the respiratory pathway (NAD metabolism/TCA cycle, pyruvate/α-ketoglutarate dehydrogenase and RCC-II), heme biosynthesis, and folate and glycine metabolism (Figure 6A). The diseases associated with components of the same module often displayed similar clinical manifestations according to the Online Mendelian Inheritance in Man (OMIM) database (Table S6). For example, porphyria is the common disorder for disease genes of the heme biosynthesis module, while glycine encephalopathy is common to diseases of the folate and glycine metabolism module. This property, of the similarity of clinical symptoms, suggests that additional proteins in these modules might be considered as candidates for diseases with related symptoms.

To discover additional features of disease susceptible genes within the mitochondrial system, we analyzed the correlation of disease orthologs with datasets on yeast deletion phenotype and orthology to proteobacteria. Among the disease orthologs, a significant enrichment of proteobacterial orthology was detected (see Figure 6B, hypergeometric test, *p* < 10^−4^). A significant correlation between NF fitness and disease gene orthology was also found: the fitness of yeast orthologs of disease genes was higher than the fitness of the remaining human orthologs (Figure 6C, *t*-test, *p* < 0.01). One explanation for the latter is that deleterious mutations in human genes among patients are possibly under-represented, because impairment of an essential gene would be lethal in humans and therefore not detected. For example, proteins of the inner membrane translocase (Tim23/Tim22 module) are essential in yeast, besides a few proteins including Tim8p, and so far, the only protein associated with a disease is the Tim8p human ortholog, DDP1, in which mutations cause the Mohr-Tranebjaerg syndrome [60].

Disease genes in our mitochondrial system are thus characterized by a tendency to have proteobacterial ancestry and a non-severe deletion phenotype in yeast. Among the top 38 proteobacterial orthologs with the least severe deletion phenotypes (fitness scores > 0.9), 26 have human orthologs and nine are known human disease genes (Table S4). Therefore, these criteria provide a resource that can be used to suggest candidate disease genes.

### Implications

We have combined established computational methods to integrate 24 large-scale datasets relevant to mitochondria to expand their parts list, and together with benchmarked measures of protein interactions place the majority of the mitochondrial proteome into a functional context. This interaction map, validated in the context of existing annotations and publicly available genome-wide datasets, covers the known mitochondrial biology more comprehensively than existing databases and recent protein complex screens [28,29]. In addition, it brings together annotated and uncharacterized proteins into a single picture which enables a survey of the entire system.

Our network yields properties of yeast orthologs to disease genes that can help in prioritizing candidate genes for human putative mitochondrial disorders. Recent studies have shown how an integrated approach to define the mitochondrial proteome in humans can identify disease genes from genomic intervals containing dozens of genes [23,61–63]. Notably, in all of these cases the disease gene was orthologous to a previously known yeast mitochondrial protein. By taking advantage of high conservation between yeast and human mitochondria, mitochondrial disease genes can be enriched by screening the orthologous yeast deletion mutants for growth patterns on respiratory and fermentative conditions (NF and F) [8]. Among the genes with human orthologs, we furthermore found that disease orthologs are more often of proteobacterial origin than would be expected by chance, which is consistent with results of an independent study [64]. Conserved proteins are often the ones at the core of the metabolic reactions of mitochondria [65], and mutations in these proteins could be critical for disease development. The fact that both properties are often observed together suggests that the subset of proteins that are conserved and have lower growth defect may be particularly susceptible to disease. It is possible that for conserved proteins that are non-essential, more variation can be present and thus a higher chance for a viable mutation phenotype may exist.

Thus, an enriched list of candidate mitochondrial disease genes can be constituted by ranking human orthologs according to whether they have a proteobacterial ortholog and a non-severe deletion phenotype in yeast. In addition, proteins in the network, and specifically proteins connected to disease genes with related clinical symptoms, may also constitute disease candidate genes.

A further implication of our network lies in extracting from global datasets the information relevant to mitochondria. Among the datasets analyzed, it had been puzzling that for most sampled conditions so far, a large set of genes were identified as differentially expressed, but when these genes were deleted—and therefore their expression forced to zero—this had no effect on fitness [55,56]. One concern was that expression change was not identifying functionally relevant genes. In contrast to genome-wide comparisons, when restricting the comparison of gene expression and deletion phenotype under NF and F conditions to only the proteins within the mitochondrial system, we find a significant positive correlation (Spearman-rank test, *p* < 0.001). This observation indicates that expression change within the mitochondrial system is likely relevant for the regulation of respiration, and suggests that systems approaches like ours may help to filter functionally relevant subsets of genes from global datasets.

Finally, our findings underscore the importance of including the extra-mitochondrial proteins related to biogenesis and function as part of the mitochondrial system. In an advancement to previous work on the mitochondrial proteome [4,10,11,16–18,20,21], we defined a mitochondrial network within its cellular context by integrating diverse datasets. Additionally, in comparison to protein networks based on interaction evidence from a single species [40,41], our network integrates data from different organisms, which is particularly useful for studying mitochondria because of their high conservation. By applying similar approaches to other cellular structures or organisms, the most advantage can be gained from integrating complementary, heterogeneous datasets. To further define the interaction map of mitochondria, new datasets that look at protein complex composition, protein localization, or regulation under different physiological conditions may prove useful. In addition, similar network strategies applied to human mitochondria have promise to accelerate the identification of genes underlying Mendelian and complex mitochondrial disorders.

## Materials and Methods

### Integration to define a mitochondrial parts list.

Two different machine learning methods were tested to predict proteins with a mitochondrial localization, namely a linear classifier (implemented by a single-layer neural network) and feed-forward Artificial Neural Networks (ANNs). All the datasets in the MitoP2 database as of January, 2005—collectively represented as a binary vector (Table S1)—were used as input for machine learning methods. These methods were trained to discriminate between 494 known mitochondrial localized proteins (reference set) and all other yeast proteins. In the case of ANNs, a single hidden layer was used, and the numbers of hidden neurons tested were: 1, 3, 5, 10, 15, 20, 25, 30, 40, 50, 60, 80, 100, 125, 150, 200, and 250. To make sure that the methods were not over-fitting, the ANNs and the linear classifier were trained in 5-fold cross validation, i.e., five copies of the predictor were trained, each using four of the five subsets for training and the remaining subset for testing. All five test set predictions were then pooled to obtain a complete set of predictions for each type of predictor, in which the score of every protein had been assigned by a predictor not trained on the protein in question. The two methods, the linear classifier (implemented single-layer neural network) and the feed-forward ANNs were benchmarked against the reference set. As shown in Figure S1, the performance of the neural networks (ANNs) is independent of the number of hidden neurons in the network and a neural network with only 1 hidden neuron (a linear model) performs as well as a network with 250 hidden neurons. Thus, there is hardly any information to be captured from correlations between the inputs, and we chose to use a linear discriminator for its simplicity and interpretability. Indeed, the linear classifier yields information on the importance of each dataset for predicting mitochondrial localization. Rather than using an ensemble of five linear discriminators (as obtained from the cross validation), we opted to train a single linear discriminator using all proteins for training. A final score for the prediction of mitochondrial proteins by the linear classifier was mapped to the [0, 1] interval with a sigmoid curve (the final scores of all proteins are available in Table S1).

The top 800 proteins with highest score were selected. This choice was based on three main reasons: first, the performance curve changes slope at 800 proteins—meaning that there is a decrease in predictive power when one goes from the top 800 to a larger number (see Figure S1A); second, the aim of the parts list prediction was to achieve high sensitivity and to capture most of the mitochondrial proteome, knowing that the network interaction analysis will later filter out proteins (indeed, the top 800 achieves 91% sensitivity and captures 451 of 494 known mitochondrial proteins); third, 800 is close to the estimated size of the mitochondrial proteome [2,4]. These reasons make the top 800 proteins a better choice for our purpose than the alternative of top 600, where an earlier break in the curve occurs. See Text S1 for further details on the choice of threshold.

### Criteria to define mitochondrial interactor candidates.

We defined mitochondrial interactor candidates as proteins having high-confidence physical interactions to the mitochondrial parts list. We selected physical protein-protein interactions from the experimental data channel (see below) and the MIPS complexes reported in STRING, having a confidence score greater than 0.85. Additionally, these proteins were filtered as having 30% of their physical interaction partners within the mitochondrial parts list. These criteria were successfully applied in a previous publication [48]. Our analysis showed that relaxing these criteria adds a large number of much more indirect physical interactors: for example, dozens of cytoplasmic ribosomal proteins (unpublished data).

### Network reconstruction.

We used the STRING database, release 6.2, to extract a functional protein network for the mitochondrial system. The choice of release 6.2 (http://string.embl.de/version_6_2) over release 6.3 is discussed in Text S1, one advantage is shown in Figure S2. In STRING each protein-protein association derives from the integration of numerous resources. The compendium of STRING evidence types for protein-protein associations includes the following channels: (1) genomic context methods (neighborhood, gene fusion, and co-occurrence); (2) co-expression of genes under a variety of experimental conditions; (3) experimental data imported from the interaction databases including BIND, DIP, GRID, HPRD, MINT; (4) curated knowledge databases such as KEGG, REACTOME, MIPS (catalog of yeast complexes), BIOCARTA (catalog of human pathways), and STKE (catalog of human signal transduction pathways); and (5) co-mentioning of genes in Medline abstracts, SGD summary paragraphs, and OMIM monographs. For all channels above, information is transferred between model-organisms where possible, using an automated setup. Essentially, interaction information is transferred between organisms when all-against-all homology searches indicated that both proteins of an interaction have likely orthologs in the other organism. If the orthology is somewhat uncertain, the evidence is transferred only partially (low scores) [38]. In our network the association is defined as “direct” if its evidence exists or is predicted in yeast or ”transferred” if it is inferred through orthology (see Table S2). A predictive confidence score is given for each association (low confidence: scores <0.4; medium: 0.4 to 0.7; high: >0.7). Our final network includes all associations from low confidence to high confidence as reported in Table S2 and Figure S4. The confidence associated with each interaction can be traced back through the hyperlinks to STRING database in Figure S4 and is also visualized in gray scale for the connecting line (dark gray = high confidence; light gray = low confidence). Protein interaction data from STRING for our network were visualized by the Medusa application [66].

### Clustering analysis.

Four hierarchical clustering algorithms were tested using the STRING confidence scores on all network associations as a similarity measure, namely single linkage, complete linkage, average linkage clustering, and a combination of single and average linkage clustering using the oc software (http://www.compbio.dundee.ac.uk/Software/OC/oc.html). Average linkage clustering with a similarity cutoff of 0.2 was chosen after systematically analyzing the size distribution of modules, the amount of known mitochondrial proteins captured in modules and manually comparing the modules to known complexes of mitochondria at multiple thresholds (described in Text S1).

### Module annotation.

To assign the functional modules, a cellular localization as well as labeling the modules, we applied the version 2.0 of the Ontologizer software (Ontologizer http://www.charite.de/ch/medgen/ontologizer). It gathers the GO annotations from all proteins and reports for each module the proportion of proteins for each GO term as well as the statistical significance of its overrepresentation. Labels and placement of modules into nucleus, mitochondria, or cytoplasm derive from combining a majority rule and a measure of the overrepresentation of a GO term for “Cellular Component” if available or of a GO term for “Biological Process” otherwise. All results are reported in Table S3.

### Comparison to annotated clusters.

We compared our modules to physical complexes as annotated in SGD (as of April 22, 2006) with GO confidence code *(TAS, IDA, IPI, NAS, IC)* and to biochemical pathways as stored in KEGG release 38.0. For each module we asked how many proteins were in common with the clusters taken from SGD and from KEGG: we defined as best match in SGD (respectively, in KEGG) the complex (respectively, pathway) that covers most of the module proteins and in case of ties, the smallest one. A module was considered recalled if its best match contains more than 50% of its components.

### Validation of candidates by in vitro synthesis of proteins and import into mitochondria.

For T7 polymerase–driven synthesis of preproteins in vitro, the open reading frames were amplified from ATG to STOP-codon by PCR, including the T7 RNA polymerase promoter and transcription initiation site within the 5′ primer. Using reticulocyte lysate (Promega, Madison, Wisconsin, United States), the resulting PCR products were utilized for coupled in vitro transcription/translation reactions to synthesize preproteins in the presence of 35S-radiolabeled methionine. Mitochondria were isolated by differential centrifugation from yeast strain W334, and mitochondrial import of synthesized preproteins was assayed as described by Ryan et al. [67].

### Analysis of deletion phenotype and gene expression under NF and F conditions.

Datasets on deletion phenotype and gene expression were obtained from the YDPM database (http://www-deletion.stanford.edu/YDPM/YDPM_index.html). For the deletion data, NF fitness was determined as the median of three non-fermentable conditions (YPL, YPG, and YPE); F fitness was measured in YPD. Cumulative distribution functions of the fitness measurements were derived for each module of five or more proteins. Inviable genes (SGD) were deemed inviable in both F and NF conditions. Genes without measurements were excluded in this analysis. The mRNA expression data were generated from log phase cultures grown under fermentable (YPD, SCD) and non-fermentable (YPL, SCL, YPE) media conditions, which in addition to yeast extract, peptone (YP) medium profiled synthetic complete (SC) medium. For every gene, we considered the following fold ratios: YPE/YPD, YPL/YPD, SCL/SCD. A stringent criterion was applied to calculate a single expression value for the difference between non-fermentable and fermentable growth: genes where the fold ratios were opposite in direction between any two conditions were set a fold ratio of 1; genes with a consistent direction of the three ratios were assigned the least-fold difference.

To assess the correlation between genes having a deletion phenotype and genes being differentially regulated we compared NF/F expression to NF-F fitness for genes in the mitochondrial system. Because the expression dataset used in this analysis received a weight close to 0 (0.003) from the linear classifier, it was not used for prediction of the mitochondrial parts list. Therefore, there is no a priori bias of the method to select genes that have both an expression change and a deletion phenotype, and this comparison is thus not subject to circular reasoning. The statistical significance of the correlation coefficient was evaluated by comparison with the distribution of correlation coefficients for shuffled ranks (Spearman rank test). See Text S1 for further discussion.

### Measurement of enzymatic activity and high-resolution respirometry.

The measurement of the specific activity of the individual respiratory chain complex IV and the synergic activity of complex II and III was performed spectrophotometrically on isolated mitochondria as described [68]. Respiration of freshly isolated mitochondria (within 1 h stored on ice) was measured at 37 °C in injection respirometers (Oroboros, Oxygraph, Innsbruck, Austria) as described [69].

### Yeast orthologs to human genes.

To assign orthology between yeast and human genes, we systematically conducted all-against-all homology searches at the level of the translated proteins, using the Smith-Waterman algorithm (substitution matrix BLOSUM62, gap open cost −11, gap extension cost −1). In order to increase sensitivity, we included in the search three additional fungal genomes, bringing the total to five genomes *(human*—Ensembl as of June, 2004, limited to longest transcript per locus; *yeast*—SPproteomes as of June, 2004; as well as *Kluyveromyces lactis, Debaryomyces hansenii,* and *Schizosaccharomyces pombe).* The homology search results were analyzed using an algorithm similar to the COGs procedure [70,71]. Briefly, the search results were first scanned for sets of proteins that are more similar within an organism than to any protein in any other organism—these were assumed to have arisen through duplication after speciation and were collected into “inparalogous groups.” Next, these groups (as well as any remaining singletons) were searched for reciprocal best matches across at least three organisms—forming a “triangle” of reciprocality. Such triangles were then allowed to seed orthologous groups. Groups were subsequently grown by including other triangles overlapping the group with at least one edge. Generally, triangles with high similarity scores were considered first, and score requirements were gradually lowered to include further triangles. As a last step, any remaining genes were included into an orthologous group if they had simple binary reciprocal matches exceeding 80 bits score (and excluding the extra fungi if necessary). The algorithm enforces all proteins in an orthologous group to have similarity to each other, thereby avoiding “domain-walking” or incompatible fragmented (pseudo)genes. All yeast and human proteins found in the same orthologous group are considered orthologs.

## Supporting Information

Figure S1 Cross-Validation and Performance Comparison of Machine Learning Approaches to Predict Mitochondrial Localized Proteins (257 KB PDF)

Figure S2 Comparison of the Scoring Schemes for Microarray Expression Data in STRING Version 6.2 and 6.3 (210 KB PDF)

Figure S3 Breakdown of the Association Evidence for Entire Network and Modules (516 KB PDF)

Figure S4 Full Network with Hyperlinks to STRING (Zoomable) (394 KB PDF)

Figure S5 Map of Modules with Five or More Proteins with Hyperlinks to STRING (Zoomable) (198 KB PDF)

Figure S6 Mapping of Expression Profiles onto the Modules Map with Hyperlinks to STRING (Zoomable) (199 KB PDF)

Table S1 Mitochondrial Parts List Prediction (1.5 KB XLS)

Table S2 Association Evidence for the Mitochondrial Network (1.6 KB XLS)

Table S3 Module Annotations (93 KB XLS)

Table S4 Analysis of Properties of the Mitochondrial System (566 KB XLS)

Table S5 Human Orthologs and Disease Gene Orthologs for the Full Yeast Genome (1.4 MB XLS)

Table S6 Disease Symptoms of Selected Modules (360 KB XLS)

Text S1 Additional Details on Methods, Findings, and Module Compositions (334 KB DOC)

## Abbreviations

F: fermentable
GO: gene ontology
KEGG: Kyoto Encyclopedia of Genes and Genomes
mtDNA: mitochondrial DNA
NF: non-fermentable
SGD: Saccharomyces Genome Database
STRING: search tool for the retrieval of interacting genes/proteins
