# Supplementary material for: Assessing Systems Properties of Yeast Mitochondria through an Interaction Map of the Organelle
Source: PLoS Genet. 2006 Oct 20;2(10):e170. doi: 10.1371/journal.pgen.0020170 (PMC1617129; doi:10.1371/journal.pgen.0020170)
Supplement: Supplementary file 1 [file pgen.0020170.sg001.pdf]

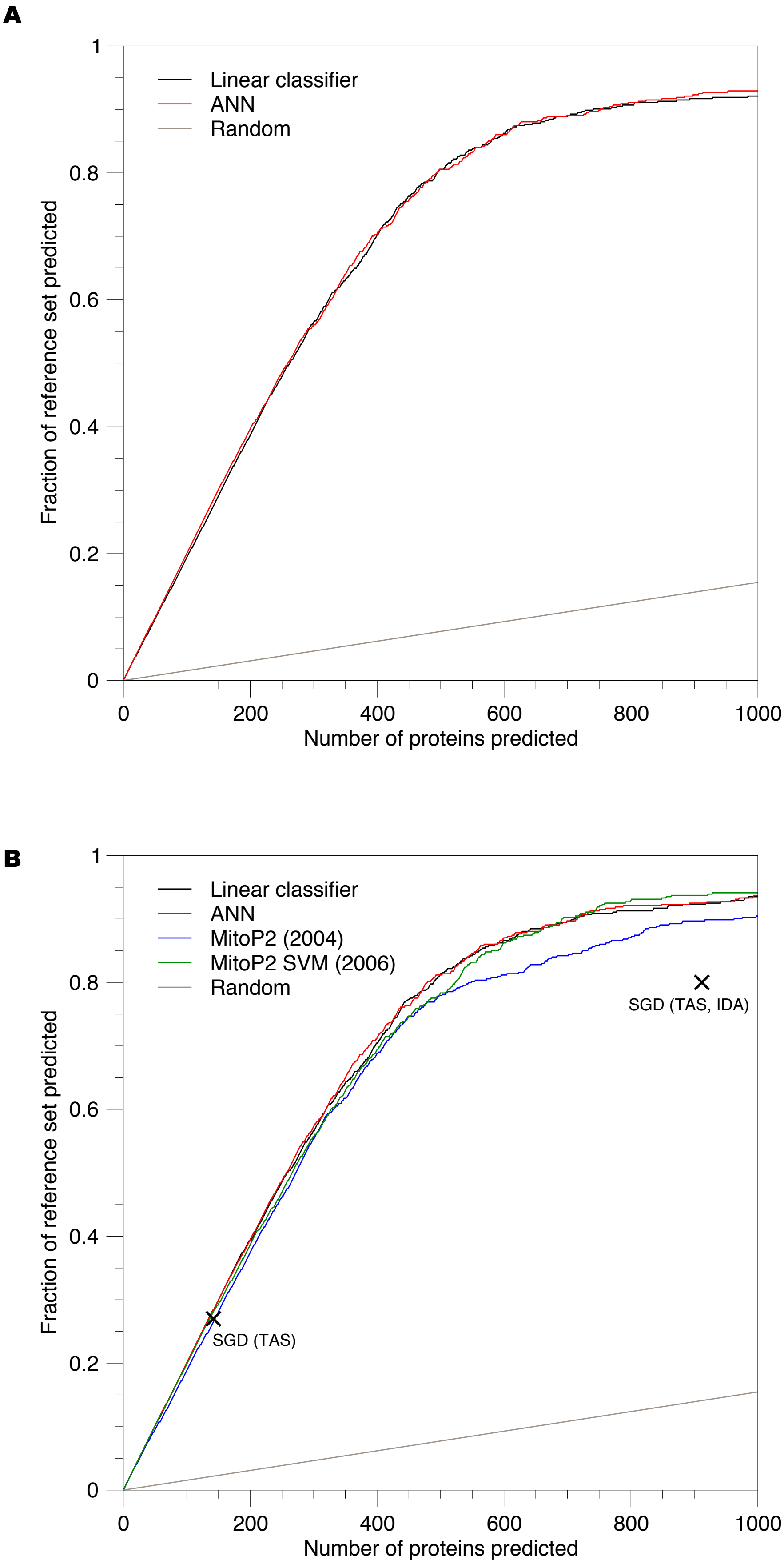

Perocchi_Figure S1

Figure S1. Cross-validation and performance comparison of machine learning approaches to predict mitochondrial localized proteins. **A,** Cross-validated performance comparison on the test set of the linear classifier and a feed-forward artificial neural network trained on 250 hidden neurons (ANN), shown in comparison to random expectation. Two different machine learning methods were tested for predicting mitochondrial proteins, namely a linear classifier and feed-forward Artificial Neural Networks (ANNs). The ANNs and linear classifier were trained in five fold cross validation, each using four of the five subsets for training and the remaining subset for testing. All five test set predictions were then pooled to obtain a complete set of predictions for each type of predictor, in which the score of every protein had been assigned by a predictor not trained on the protein in question**.** For each of two scoring schemes, the fraction of the reference set recalled (sensitivity) is plotted as a function of the number of positive predictions made. No substantial performance improvement was obtained by using artificial neural networks (shown here with using 250 hidden neurons) rather than a linear classifier. **B,** Comparison of training set performance of the linear classifier, feed-forward artificial neural network (ANN with 250 hidden neurons), MitoP2 predictions from Prokisch et al. 2004 [1], and MitoP2 predictions by the SVM approach [2], shown in comparison to SGD gene ontology annotations and random expectation**.** The linear classifier performed substantially better than the original MitoP2 algorithm published by Prokisch et al. 2004 [1] when applied to the same 24 datasets, and at least as good as the more recent MitoP2 predictions by the SVM approach [2]. For comparison, we also plot the proteins having in SGD a gene ontology annotation for a mitochondrial cellular compartment with the most stringent evidence codes: with TAS (Traceable

Author Statement) and with TAS or IDA (Inferred from Direct Assay). This comparison shows that all machine learning approaches outperform the annotation in SGD.

References:

1. Prokisch H, Scharfe C, Camp DG, 2nd, Xiao W, David L, et al. (2004) Integrative analysis of the mitochondrial proteome in yeast. PLoS Biol 2: e160.
2. Prokisch H, Andreoli C, Ahting U, Heiss K, Ruepp A, et al. (2006) MitoP2: the mitochondrial proteome database--now including mouse data. Nucleic Acids Res 34: D705-711.
