# Supplementary material for: Assessing Systems Properties of Yeast Mitochondria through an Interaction Map of the Organelle
Source: PLoS Genet. 2006 Oct 20;2(10):e170. doi: 10.1371/journal.pgen.0020170 (PMC1617129; doi:10.1371/journal.pgen.0020170)
Supplement: Supplementary file 2 [file pgen.0020170.sg002.pdf]

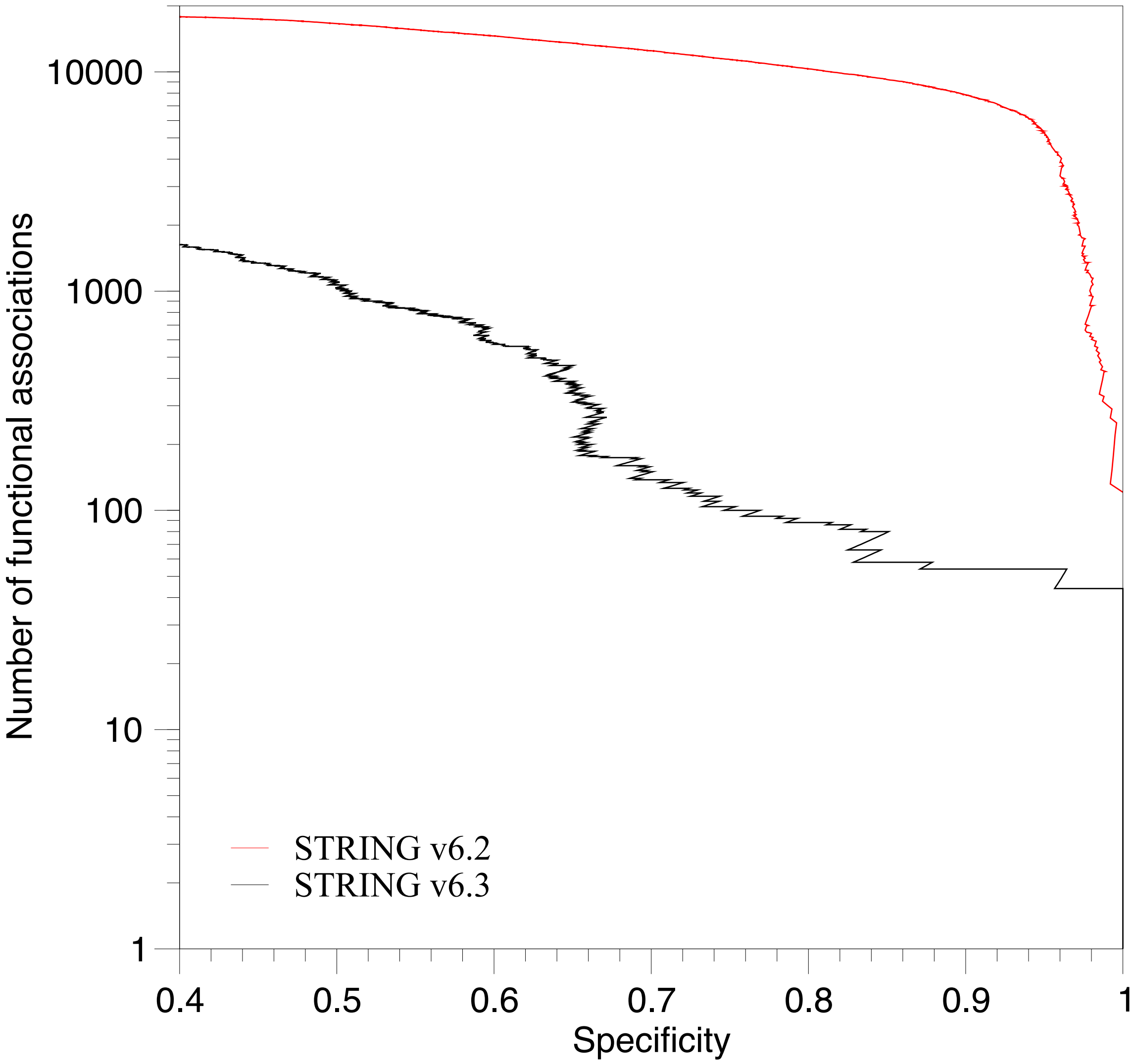

Perocchi_Figure S2

Figure S2. Comparison of the scoring schemes for microarray expression data in STRING version 6.2 and 6.3. The number of functional interactions proposed for yeast proteins is plotted as function of the specificity. The latter was calculated as the fraction of predicted interactions between proteins in KEGG that belong to the same KEGG metabolic map [1]. Compared to this reference, the STRING version 6.2 scoring scheme performs far better than that of STRING version 6.3. It should be noted that the scoring scheme in STRING version 6.2 performs well for metabolic proteins, since the scoring scheme was trained on these. This scoring scheme was changed in version 6.3 because it was too computationally intensive to handle the growing amount of expression data, and also because it may perform less well on non-metabolic proteins and on proteins from other organisms than suggested by this benchmark. Since the mitochondrial system is dominated by metabolic proteins, the STRING version 6.2 scoring scheme is so far the most appropriate for constructing a network of yeast mitochondria.

References:

1. Jensen LJ, Lagarde J, von Mering C, Bork P (2004) ArrayProspector: a web resource of functional associations inferred from microarray expression data. Nucleic Acids Res 32: W445-448.
