# Supplementary material for: Assessing Systems Properties of Yeast Mitochondria through an Interaction Map of the Organelle
Source: PLoS Genet. 2006 Oct 20;2(10):e170. doi: 10.1371/journal.pgen.0020170 (PMC1617129; doi:10.1371/journal.pgen.0020170)
Supplement: Supplementary file 3 [file pgen.0020170.sg003.pdf]

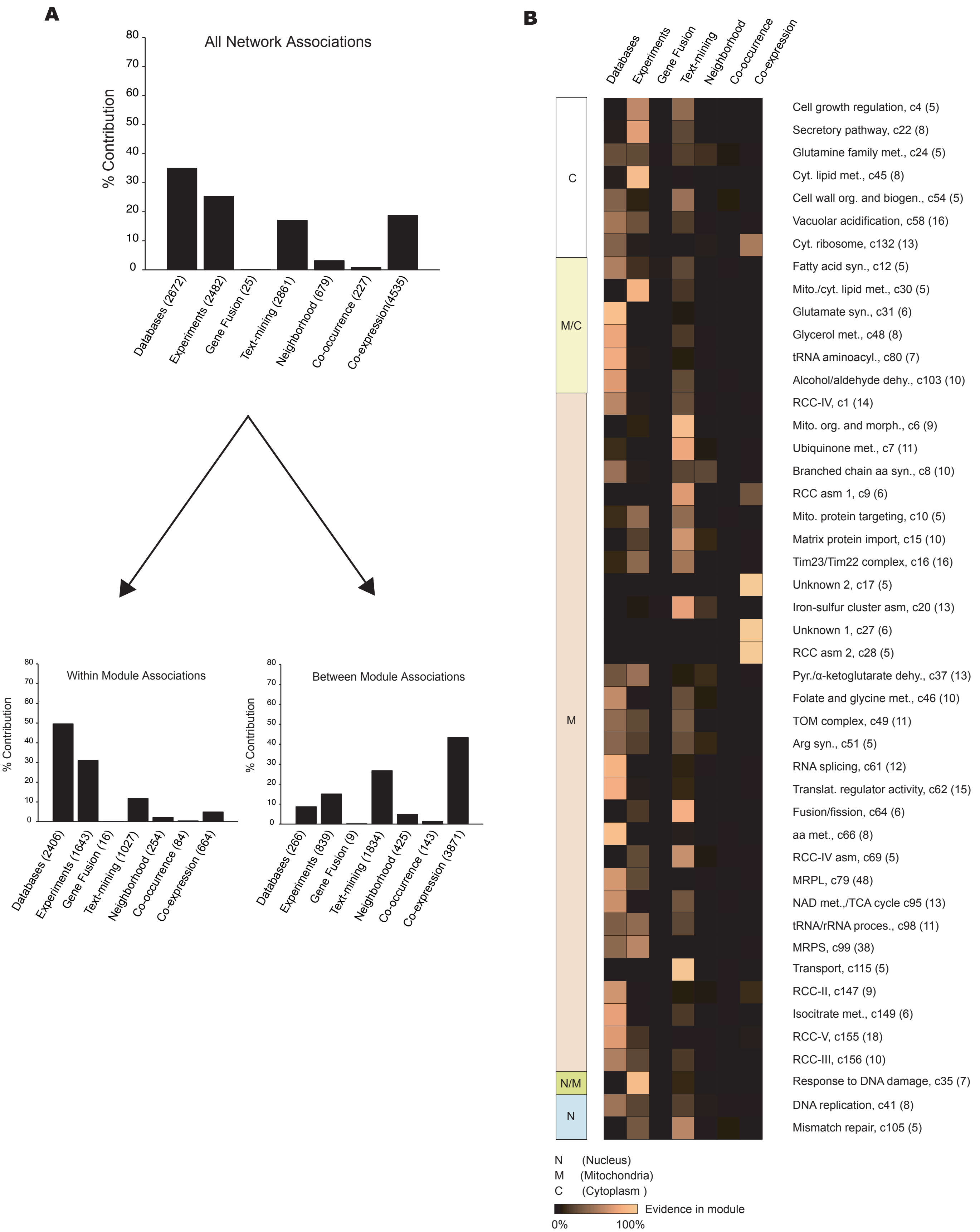

Perocchi_Figure S3

Figure S3. Breakdown of Association Evidence for Entire Network and Modules. **A**, Each protein-protein association has an evidence score associated with it. The percent contribution of each evidence type is plotted for the entire network and for each subcategory of associations: within module associations and between module associations. In parenthesis the total number of associations is given. **B**, The percent contribution of each evidence type to the total evidence in each module is plotted using a color scale. The modules are assigned to localization categories. For each module the module number is indicated preceded by ‘c’ for ‘cluster’ and in parentheses the number of proteins in the module.
