# Supplementary material for: Assessing Systems Properties of Yeast Mitochondria through an Interaction Map of the Organelle
Source: PLoS Genet. 2006 Oct 20;2(10):e170. doi: 10.1371/journal.pgen.0020170 (PMC1617129; doi:10.1371/journal.pgen.0020170)

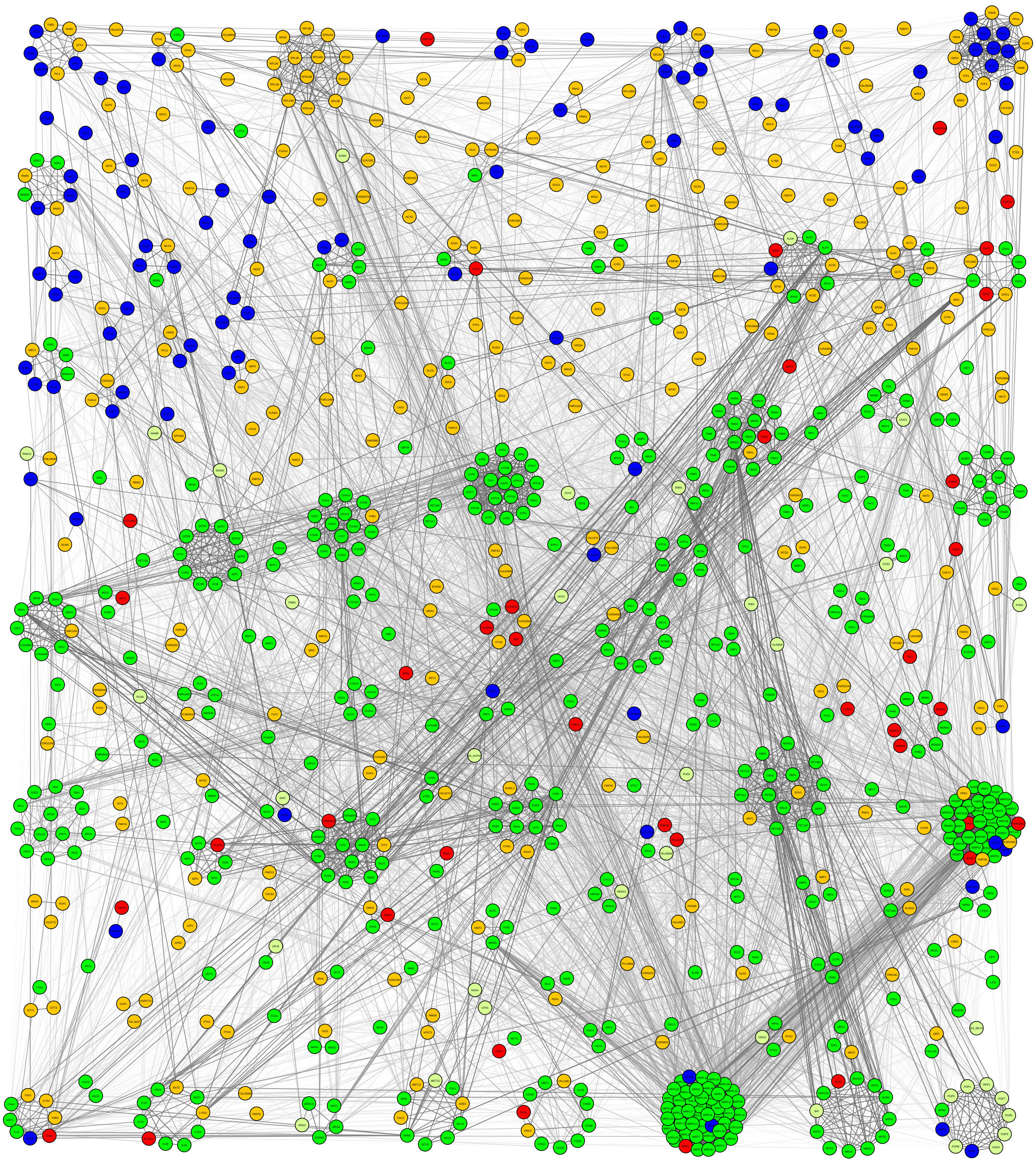

Supplement: Supplementary file 4 [file pgen.0020170.sg004.pdf]
