# Supplementary material for: Assessing Systems Properties of Yeast Mitochondria through an Interaction Map of the Organelle
Source: PLoS Genet. 2006 Oct 20;2(10):e170. doi: 10.1371/journal.pgen.0020170 (PMC1617129; doi:10.1371/journal.pgen.0020170)
Supplement: Supplementary file 5 [file pgen.0020170.sg005.pdf]

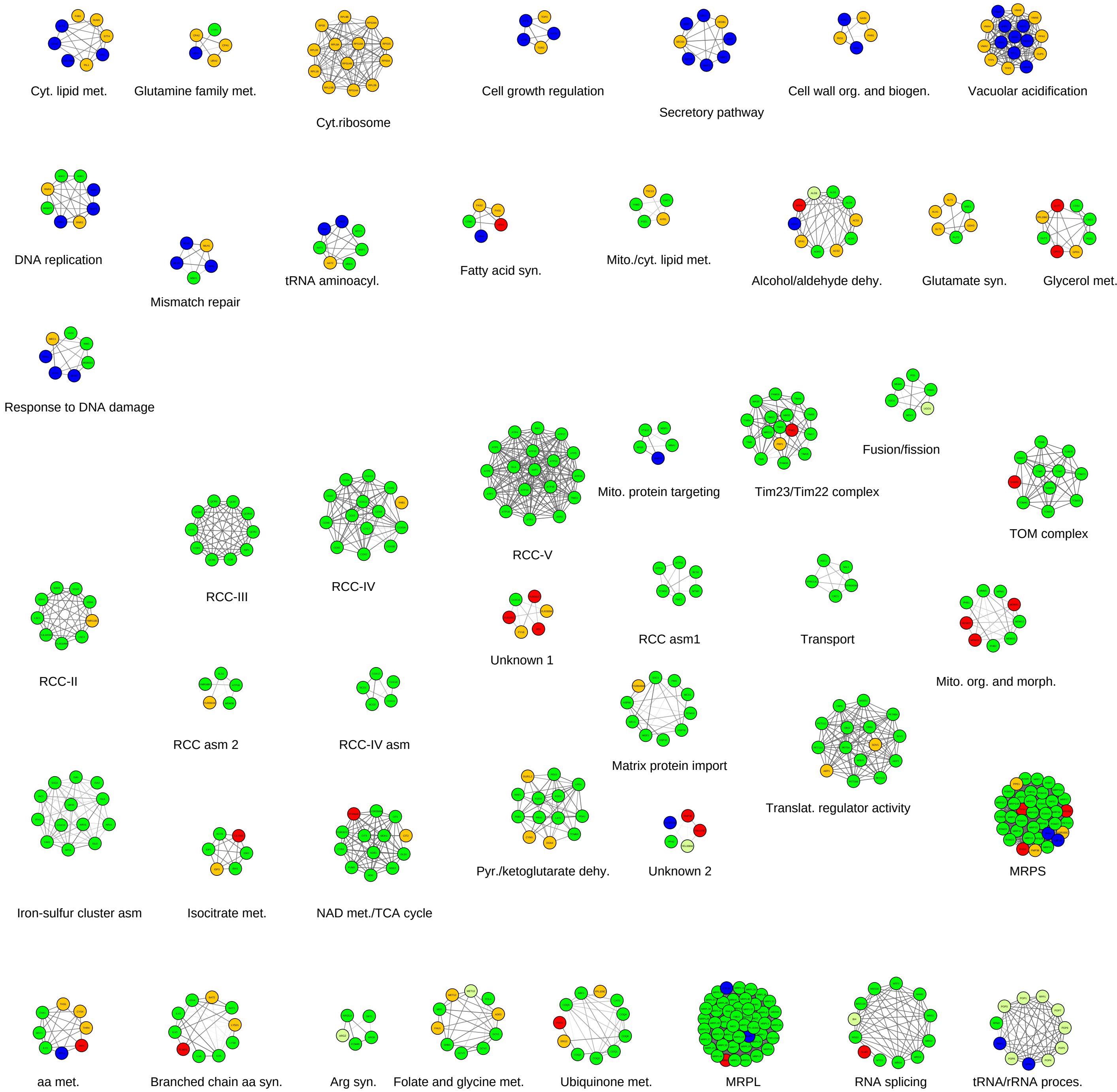
Cyt. lipid met.
Glutamine family met.
Cyt.ribosome
Cell growth regulation
Secretory pathway
Cell wall org. and biogen.
Vacuolar acidification
DNA replication
Mismatch repair
tRNA aminoacyl.
Fatty acid syn.
Mito./cyt. lipid met.
Alcohol/aldehyde dehy.
Glutamate syn.
Glycerol met.
Response to DNA damage
Fusion/fission
Tim23/Tim22 complex
Mito. protein targeting
RCC-V
TOM complex
RCC-III
RCC-IV
RCC-II
Unknown 1
RCC asm1
Transport
Mito. org. and morph.
RCC asm 2
RCC-IV asm
Matrix protein import
Translat. regulator activity
Pyr./ketoglutarate dehy.
Unknown 2
MRPS
Iron-sulfur cluster asm
Isocitrate met.
NAD met./TCA cycle
aa met.
Branched chain aa syn.
Arg syn.
Folate and glycine met.
Ubiquinone met.
MRPL
RNA splicing
tRNA/rRNA proces.
