# Supplementary material for: Assessing Systems Properties of Yeast Mitochondria through an Interaction Map of the Organelle
Source: PLoS Genet. 2006 Oct 20;2(10):e170. doi: 10.1371/journal.pgen.0020170 (PMC1617129; doi:10.1371/journal.pgen.0020170)
Supplement: Supplementary file 7 [file pgen.0020170.sd001.doc]

**Supplementary Notes**

**Assessing systems properties of yeast mitochondria through an interaction map of the organelle**

Fabiana Perocchi^1^, Lars Juhl Jensen^1^, Julien Gagneur^1^, Uwe Ahting^2^, Christian von Mering^1^, Peer Bork^1^, Holger Prokisch^2^, Lars M. Steinmetz^1*^

**1** European Molecular Biology Laboratory, Meyerhofstrasse 1, Heidelberg, Germany

**2** Institute of Human Genetics, Technical University Munich and GSF National Research Center for Environment and Health, Munich, Germany

**Setting of cut-offs**

The approach taken to derive a set of functional modules is described in Figure 1 and consists of three main computational steps. **First**, a simple linear classifier is used to integrate 24 genome-wide datasets and assign a likelihood of mitochondrial localization to every gene product in the yeast genome. **Second**, STRING is used to extract physical protein-protein associations. **Third**, hierarchical mean-clustering algorithm is applied to derive modules of functionally connected proteins. Each of the above steps requires the selection of a score threshold, dependent on the application and on evaluation of sensitivity and specificity.

**1. Parts list prediction**

A scoring scheme for predicting a comprehensive and reliable catalog of mitochondrial proteins was developed using machine learning methods. We integrated 24 genome-wide datasets on mitochondrial localization, phenotype and regulation which provide complementary clues about mitochondrial localization and regulation (Table S1). A compendium of the datasets is listed below. Most of them attempt to directly identify or indirectly predict proteins with a mitochondrial localization. Through the use of a localization list as a training set, we aim to enrich our candidate list for mitochondrial localized proteins. Nevertheless the inclusion of functional datasets such as expression profiles and deletion phenotype screenings allows the prediction of proteins that may localize outside mitochondria but affect mitochondrial function and biogenesis. We explored two computational methods (ANNs and linear classifier) for integrating features provided by the 24 genome-scale datasets and compared to published algorithms [1,2]. As shown in Figure S1, machine learning algorithms (ANNs, simple linear classifier, SVM) perform better than other methods [1] and in comparison to trustable (TAS) and high-throughput (IDA) annotations for mitochondrial proteins in the SGD database. We find the performance of the neural networks (ANNs) to be independent of the number of hidden neurons in the network. A neural network with only 1 hidden neuron (a linear model) performs as well as a network with 250 hidden neurons. This can be explained as there is hardly any information to be captured from correlations between the inputs. Thus the [1] scoring scheme is more complex than needed since it tries to capture correlations among datasets. As no significantly higher sensitivity is reached from the SVM algorithm (Figure S1) we opted to use a simple linear classifier which has the advantage that the predictive power of each dataset is apparent. With the linear classifier the likelihood of mitochondrial localization of every gene product in the yeast genome was computed. The proteins are ranked from higher to lower prediction scores and reported in Table S1, allowing different cutoffs to be applied. Here, we use a threshold higher than 0.413 based on the performance comparison plot (Figure S1), corresponding to a sensitivity of 91%: At this threshold, the linear discriminator correctly predict 451 of the 494 known mitochondrial proteins (training set) as well as an additional 346 proteins not in the training dataset.

The choice of the top 800 is based primarily on four reasons: (1) we wanted to achieve high sensitivity and capture most of the mitochondrial proteome, knowing that the network interaction analysis will later filter out additional proteins; (2) the performance curve (Figure S1) changes slope at 800 proteins -- meaning that there is a marked decrease in predictive power when one goes from the top 800 to a larger number; (3) at 800 we capture almost all of the known mitochondrial localized proteins; and (4) 800 is the predicted number of mitochondrial localized proteins from proteomic studies. All of these reasons make 800 a good choice.

An earlier break occurs in the top-600, thus the difference between the set of top-800 proteins and the top-600 proteins was further evaluated for protein composition. The set of the 200 proteins includes 23 reference set proteins and 174 candidates (three proteins annotated in the SGD database as hypothetical and derived from dubious ORFs were omitted). The latter were manually inspected through a literature based evaluation of their plausible involvement in mitochondrial functions. Their connectivity was also evaluated based on the resulting network (see Figure S4). Most of these candidates define extra-mitochondrial localized modules, but with functional connections to mitochondria as documented for the candidates of the vacuolar acidification module (more below). Other five candidates have recently been annotated in the MitoP2 database (release January 2005) as mitochondria-localized proteins according to single-gene experimental verification. Our incentive was to capture mitochondrial associated proteins that localize outside the organelle, but could be involved in the interface between mitochondria and the outside compartments. We expected that proteins with scores following those of most mitochondrial localized proteins would be enriched for proteins functionally associated with the organelle. All of these reasons make the set of top-800 a better choice for our purpose than the set of top-600.

**2. Interactor candidates**

A system-level definition of the mitochondrial organelle needs to take into account the tight integration of its proteome with the cellular context. In addition to the set of proteins that constitutively reside within the mitochondria, there are proteins that transit and are transiently interacting with the organelle. Several cytoplasmic and nuclear proteins have been reported as having also a mitochondrial localization and vice versa several known mitochondrial proteins play additional roles outside the organelle. In order to expand the mitochondrial proteome we additionally extract for the total of 840 proteins, high confidence physical protein-protein associations from the “experiments” channel and the MIPS complexes in STRING version 6.2 (see a description of STRING association evidence types below). Although a dataset of physical protein interactions reported in von Mering et al. [3] was one of the large-scale datasets integrated by the linear discriminator, this dataset represents only a subset of the protein-protein interactions defined by the “experiments” channel and the MIPS complexes now integrated in the version 6.2 of STRING. Additional proteins confidently interacting with the above set were thus identified by searching for proteins for which at least 30% of its physical interaction partners at a confidence level of 0.85 are in the above 840 proteins, adding 95 further proteins to the parts list. These criteria for defining the 95 high-confidence physical interactors are the same as successfully used for an analogous purpose by a previous publication [4] and therefore we have kept these settings. Our analysis shows that relaxing these criteria adds a large number of much more indirect physical interactors: for example dozens of cytoplasmic ribosomal proteins. We tested different criteria and chose to be stringent to limit our analysis to interactors for which strong experimental evidence exists.

**3. Clustering**

Hierarchical clustering was done on pairwise functional associations. We tested four different hierarchical clustering methods (single linkage, complete linkage, average linkage, and a combination of single and average linkage clustering) and carefully evaluated different cutoffs for each clustering method.

As expected complete linkage clustering was too stringent and single linkage clustering too lenient. Average linkage clustering gave good results and was chosen because it integrates all association information in our network. Cutoffs were varied from 0.05 to 0.95 in 0.05 interval steps. For each cutoff, we systematically analyzed the size distribution of modules and the coverage of known mitochondrial proteins in modules. We also manually compared the modules to known complexes of mitochondria. One criteria for this manual comparison was that each respiratory chain complex (II—V) should be assembled into a single, distinct cluster and they should not merge to form a single super-complex. The table below shows cluster sizes resulting from three cutoffs. On these cutoffs we also tested whether filtering on single linkage with different threshold would help to ‘clean up’ the network after average linkage clustering. However single linkage filtering (at threshold 0.3) combined with average linkage clustering mainly had the effect of breaking clusters consisting of a pair to singletons but did not have much effect on reducing clusters of 5 or more proteins (see table). We therefore applied average linkage clustering alone. Analysis of the resulting clusters at multiple thresholds as described above narrowed the search for average linkage clustering to 0.2 as a valid choice for the purpose of our study.

The choice of the clustering threshold of 0.2 is less crucial since our mitochondrial network consists of all the interactions in the full network prior to clustering (Figure 3A, Figure S4) and the cutoff for defining modules is mainly used to break the full network into modules which are small enough to be analyzed.

| Method | Cutoff for average linkage | Clusters (pairs or more) | Clusters (five or more) | Proteins in clusters of pairs or more | Proteins in clusters of five or more | Proteins of the 935 not captured in clusters of five or more |
| --- | --- | --- | --- | --- | --- | --- |
| Average linkage clustering | 0.1 | 125 | 53 | 810 | 599 | 336 |
|  | 0.15 | 149 | 54 | 796 | 549 | 386 |
|  | **0.2** | **164** | **46** | **783** | **474** | **461** |
| Average linkage plus single linkage filtering (0.3) | 0.1 | 118 | 52 | 786 | 594 | 341 |
|  | 0.15 | 139 | 54 | 776 | 549 | 386 |
|  | 0.2 | 155 | 46 | 765 | 474 | 461 |

In bold the method we chose.

**Choice of STRING version 6.2.**

Both the current release of STRING (version 6.3, December 2005 at <http://string.embl.de/>) and the previous release (version 6.2, June 2005 at <http://string.embl.de/version_6_2/>) were considered to derive a functional network of yeast mitochondria. The primary difference between the current and the previous releases consists in the scoring scheme applied to integrate expression datasets and only minor improvement of the textmining algorithm. Figure S2 shows a comparison of the scoring schemes for microarray expression data in STRING version 6.2 and 6.3 where the number of functional interactions proposed for yeast proteins is plotted as function of the specificity. The STRING version 6.2 scoring scheme performs better than that of STRING version 6.3. Moreover, when tested on metabolic pathways in yeast, the old scoring scheme performs better than the new one since the scoring scheme was trained on metabolic proteins [5]. Since the mitochondrial system is dominated by metabolic proteins, we hence opted to use the STRING version 6.2 scoring scheme as most appropriate for constructing a network of yeast mitochondria. Overall only few modules defined as co-regulated genes would be affected and isolated proteins exclusively linked by textmining evidence.

**Statistical analysis on the mitochondrial system -- avoiding circularity**

1. The set of human orthologs was used to calculate the degree of conservation of the mitochondrial system to humans (Table S5). Analysis of conservation to humans yielded orthologs for 60% (565/935) of the proteins in the yeast mitochondrial system which is significantly higher (hypergeometric test, P <10^-21^) than the conservation found for the entire yeast proteome (46%). To avoid circularity in this analysis, since conservation to humans was used as criteria to define the parts list, from the calculation we excluded 29 primary candidate proteins having a human ortholog with mitochondrial localization (see dataset in Table S1) and the 95 interactor candidates, since inter-species transfer is used in STRING to predict associations. The resulting subset counted 811 proteins of which 479 still have a human ortholog according to the algorithm used. When excluding all proteins from the parts list that could have been included in part due to orthology to humans, the conservation of the yeast system to humans remains significant (corrected P-value, Hypergeometric test, P < 10^-15^).

2. To assess the correlation between genes having a deletion phenotype and genes being differentially regulated we compared NF/F expression to NF-F fitness for genes in the mitochondrial system. Because the expression dataset used in this analysis received a weight close to 0 (0.003) from the linear classifier, it was *de facto* not used for prediction of the mitochondrial parts list. Thus a comparison of the two datasets over the mitochondrial system does not lead to circular reasoning. The statistical significance of each correlation coefficient was evaluated by comparison with the distribution of correlation coefficients for shuffled ranks (Spearman rank test). All the genes annotated as inviable in SGD were not considered in this calculation.

3. The set of disease gene orthologs was not used for predicting the mitochondrial parts list. Furthermore, the correlation between disease gene orthologs and genes with a proteobacterial origin, and the correlation between disease gene orthologs and genes with a deletion phenotype in yeast, were calculated within the set of genes in the mitochondrial system that is conserved between yeast and humans (human orthologs). Among this set of genes, the disease genes were found to be more often of proteobacterial origin. Furthermore we find a correlation of disease genes with lower deletion phenotypes, opposite to what a bias in the prediction phase of the algorithm would lead to. These observations thus cannot be explained by the fact that mitochondria are highly conserved among eukaryotes or by circularity coming from the prediction phase of the parts list.

**Comparison to two large-scale screens of protein complexes**

Two recent studies [6,7] provide the most comprehensive screens for protein complexes in yeast. Altogether about 76% of the yeast proteins were identified and half of the proteome was reported in complexes. Thus we compared these datasets to our mitochondrial module map. We find that only about 100 proteins of the 30 mitochondrial localizing modules in our network could be captured by Krogan *et al.* or Gavin *et al.* and respectively, 38 and 60 of the about 100 proteins, are part of the mitochondrial ribosomal subunits. Even though these studies provide invaluable data on the set of complexes for the entire yeast cell, they alone do not cover the mitochondrial proteome which includes many membrane protein complexes and is dependent on specific growth conditions.

**Compendium of MitoP2 datasets that were integrated**

The following types of complementary large-scale data were downloaded from MitoP2 (<http://ihg.gsf.de/mitop2/> as January 2005; number of datasets given in parenthesis):

*Mutant phenotype* (2). Mutants are screened for growth defects under conditions where mitochondria are active [8,9]. Benefits: it is a genetic approach that finds functionally important proteins both inside and outside the organelle. Drawbacks: redundant genes are not detected as only single genes are analyzed; requires a mutant collection.

*Subcellular localization* (2). Microscopy-based localization of tagged proteins [10,11]. Benefits: localization in vivo. Drawbacks: tagging of proteins may alter localization.

*Proteomics* (7). Analysis of mitochondrial purified proteins using mass spectrometry [1,12-14], or analyzing proteins that physically interact with known mitochondrial proteins [3,15-18]. Benefits: direct measurement for localization or interaction. Drawbacks: biased towards highly abundant proteins.

*Transcriptome* (4). mRNA expression analysis under conditions predictive for mitochondria [1,19-21]. Benefits: easy to apply genome-wide. Drawbacks: mRNA expression changes often do not correlate with function or localization.

*In silico prediction of mitochondrial localization* (4). Mitochondrial targeting signal sequences [22,23] and differences in e.g. amino acid composition between mitochondrial and other proteins [24-26]. Benefits: easy to apply genome-wide. Drawbacks: many mitochondrial proteins lack signal sequences; only predicts proteins localized to the organelle.

*Homology* (5). Sequence homology to known mitochondrial proteins in other species [27], to the alpha-proteobacteria Rickettsia prowazekii [28], or absence of homology to Encephalitozoon cuniculi that lacks mitochondria [29]. Benefits: easy to apply genome-wide. Drawbacks: limited to conserved proteins.

**Compendium of STRING evidence types for protein-protein associations**

*Genomic context methods (neighborhood, gene fusion and co-occurrence)*: Benefits: computational methods which become increasingly accurate as more genomes are sequenced. Drawbacks: implies only functional associations; primarily restricted to proteins with bacterial orthologs.

Neighborhood: Genes frequently found in genomic vicinity (having the same orientation, and being observed in 'runs' with intergenic distances of less than 300 bp)

Gene fusion: Genes found fused (encoding a single open reading frame) in other organisms

Co-occurrence: Genes with a similar profile of occurrence in the various species ('phylogenetic profile', 'presence-absence profile'). Pairs of homologs are largely excluded from this channel.

*Co-expression*: Genes found co-expressed under a variety of experimental conditions. Benefits: many datasets available. Drawbacks: associations often indirect.

*Experiments*: imported data from interaction databases (redundant entries are considered only once) such as BIND - DIP - GRID - HPRD - MINT.

*Databases*: imported associations from curated knowledge catalogs. Databases: KEGG - REACTOME - MIPS (catalog of yeast complexes) - BIOCARTA (catalog of human pathways). Benefits: high confidence-curated evidence for physical interaction. Drawbacks: limited availability; might be highly biased towards current knowledge.

*Text-mining*: Co-mentioning of genes (using various aliases) in PubMed-Abstracts. Benefits: computational search of a large data resource. Drawbacks: association type is not specified.

*Inter-species Transfer*: for all channels above, information was transferred between model-organisms where possible, using an automated setup. Essentially, interaction information was transferred between organisms when all-against-all homology searches indicated that both proteins of an interaction have likely orthologs in the other organism. If orthology is somewhat uncertain, transfer is only partial (low scores).

**Details on individual modules (the module number is given in parenthesis)**

A description of the components of most functional modules is given together with the association evidences that group them in the same module and an explanation for possible inclusion of novel mitochondrial candidates. The latter remain to be proven.

**Mitochondria-localized modules**

**tRNA/rRNA processing (c98).** This module includes components of two main physical units: the RNase P and RNase MRP together with several proteins involved in pre-rRNA processing. Some of the components are annotated as shared among these sub-complexes explaining the link between them. RNase mitochondrial RNA processing (MRP) is an essential ribonucleoprotein endoribonuclease that has been shown to cleave mitochondrial primer RNA sequences from a variety of sources [30]. It consists of at least nine protein components. Most of the RNase MRP activity is found in the nucleolus where it plays a role in the processing of 5.8S rRNA. Eight of the nine components are shared with RNase P, a complex involved in processing of tRNA precursors. Both RNase P and MRP localize to the nucleolus and the mitochondria and have been shown to cleave common substrates.

**MRPS (c99), MRPL (c79).** The mitochondrial small (MRPS) and large (MRPL) ribosomal subunits include structural constituents of mitochondrial ribosomes. Mitochondria contain their own translation machinery [31]. Mitochondrial ribosomes do not share any constituents with the cytoplasmic ones, although all but one protein are encoded by the nuclear genome, synthesized on cytoplasmic ribosomes and subsequently imported into the mitochondria. Primary candidates in the MRPS module have strong evidences of a mitochondrial localization from large-scale studies and computational prediction of mitochondrial signal peptides and show a growth defect on respiratory carbon sources. Interestingly, the mitochondrial localization of the primary candidate Ygl068wp, in the MRPL module, was recently confirmed by single gene studies [32]. Proteins of both MRPS and MRPL do not appear to be well conserved. The majority of the MRPs whose sequences are known are completely unique, having a relatively low degree of sequence conservation [31]. It has been speculated that the conserved proteins might carry out the central step of protein synthesis, while additional proteins have more specialized functions in coupling of the translation process to the mitochondrial inner membrane. The MRPS and MRPL modules show a significant growth defect when deletion mutant in their components are grown in NF conditions if compared to fermentable ones. This is consistent with their role in translating core components of the respiratory chain complexes.

**NAD metabolism/TCA cycle (c95) and Isocitrate metabolism (c149).** The module NAD metabolism/TCA cycle includes well known mitochondrial enzymes involved in NADH oxidation and Krebs cycle. The protein Ndi1p functions as a rotenone-insensitive matrix-facing NADH-ubiquinone oxidoreductase [33]. In addition to matrix NADH, yeast mitochondria are able to oxidize cytosolic NADH via an intermembrane space-facing NADH-ubiquinone oxidoreductase, an activity supported by at least *NDE1* and *NDE2* genes. The Krebs cycle is a well known pathway involved in the second stage of cellular respiration and is carried out in the mitochondrial matrix. Two primary candidates, Ypr004cp and Cit2p have evidences of mitochondrial localization. Cit2p is involved in the glyoxylate cycle as peroxisomal citrate synthase, but it has been reported to have a key role in retrograde regulation [34]. Most of the components of the TCA cycle module are up-regulated during growth on respiratory carbon sources, but only few of them show a respiratory-specific deletion phenotype. The module involved in isocitrate metabolism contains enzymes that are either involved in the synthesis (Aco1p and Yjl200cp) or in the catabolism of isocitrate (Idh1p, Idh2p and Idp3p). The primary candidate, Yjl200cp, is a hypothetical protein with multiple evidence of mitochondrial localization from large-scale and high-throughput datasets. Idh1p and Idh2p are two NAD(+)-dependent isocitrate dehydrogenases, which catalyze the oxidation of isocitrate to alpha-ketoglutarate in the TCA cycle. Instead Idp1p is a mitochondrial NADP-specific isocitrate dehydrogenase that catalyzes the oxidation of isocitrate to alpha-ketoglutarate to be used in biosynthetic processes. The primary candidate, Idp3p, is a peroxisomal NADP-dependent isocitrate dehydrogenase that catalyzes the oxidation of isocitrate required for growth on unsaturated fatty acids. Proteomic studies on purified mitochondria and screening of mRNA found on mitochondrial-bound polysomes suggest for Idp3p a mitochondrial location. The expression of *IDP3* is up-regulated on respiratory conditions.

**RCC-II (c147).** This module includes proteins with succinate dehydrogenase and succinate-CoA ligase activity. The oxidation of succinate is coupled to the transfer of electrons to ubiquinone by the respiratory chain complex II. The expression of all genes in this module increase during respiratory metabolism and deletion mutants have a severe fitness defect under these conditions. All of the components of RCC-II are encoded by the nuclear genome. The functional module is well conserved to alpha-proteobacteria and humans. Mutations in the orthologous human genes are associated with mitochondrial disorders.

**RCC-III (c156).** The ubiquinol-cytochrome c oxidoreductase (RCC-III) is a respiratory chain complex composed of nine subunits of which only one is encoded by the mitochondrial genome, Cobp. The expression of these subunits is significantly up-regulated during growth on NF carbon sources where they also show a respiratory-specific deletion phenotype. Core components of RCC-III have a detectable proteobacterial orthology and are also conserved to humans.

**RCC-IV (c1)**. This module includes structural subunits of the cytochrome c oxidase complex (RCC-IV). The three catalytic core subunits, Cox1p, Cox2p and Cox3p are encoded by mitochondrial genes. These genes show proteobacterial origin. Other nine proteins are accessory subunits of this complex. The primary candidate, Yhb1p, is a nitric oxide oxidoreductase; four independents proteomic assays on purified mitochondria suggest for it a mitochondrial localization. Indeed a recent study localizes Yhb1p in both the cytosol and the mitochondrial matrix of respiring cells [35]. The linkages of Yhb1p to Mrpl9p and Pet54p are due to a textmining artifact (gene names co-mentioned in a sequencing paper). YHB1 clusters in RCC-IV through its connection with COX5B and CYC7, this connection is based on textmining, but not an artifact. The module shows a significant respiratory-specific deletion phenotype on NF carbon sources and almost all components are up-regulated at the mRNA level. As reported in a single gene study, *COX5B* levels are low during aerobic growth, showing an inverse regulation with *COX5A* by oxygen and heme [36].

**RCC-V (c155).** The ATP synthase complex (RCC-V) includes subunits encoded by nuclear genes and by mitochondrial genes. Three subunits are mitochondrial encoded: Aap1p, Oli1p and Atp6p. The ATP synthase couples oxidative phosphorylation with the generation of ATP. With the exception of some assembly factors [37] the structure of ATP synthase is highly conserved among diverse organisms and consists of two major components, the soluble F1 and membrane-bound F0 [38]. The catalytic core of the enzyme resides in the F_1_ component and consists of a hexamer of alternating alpha and beta subunits (Atp1p and Atp2p). Both of these components have a detectable proteobacterial ancestry and are conserved to humans. F_1_ and F_0_ are connected, both functionally and physically, via two additional multi-subunit structures, the central stalk and the stator stalk. The gamma subunit, Atp3p is a component of the central stalk while Atp4p and Atp5p are subunits of the stator stalk, a stationary structure necessary for the productive transmission of rotary motion from the F_0_ proton pump to the F_1_ catalytic core. The F_0_ proton pore is formed by a ring of twelve Oli1p subunits. Atp6p is a component of F_0_ and provides a stationary structure necessary for the productive rotation of Oli1p.

**Pyruvate/α-ketoglutarate dehydrogenase (c37).** This module includes two main physical complexes: the pyruvate dehydrogenase complex (PDH) and the alpha-ketoglutarate dehydrogenase complex (KGD). These complexes are physically connected through Lpd1p that is a multifunctional enzyme that participates in both complexes. The PDH complex [39] is a large multicomponent enzyme complex that catalyzes the conversion of pyruvate to acetyl-CoA. It is composed of a pyruvate dehydrogenase (component E1 – Pda1p and Pdb1p), a dihydrolipoamide acetyltransferase (component E2 – Lat1p), and lipoamide dehydrogenase (component E3 – Lpd1p). A fifth subunit (Pdx1p) of the pyruvate dehydrogenase complex links Lat1p to Lpd1p. In contrast to the E1 and E2 subunits which are complex-specific, the E3 subunit is also part of 2-oxoglutarate dehydrogenase and branched-chain 2-oxoacid dehydrogenase. It has been shown that the expression of the PDH complex is independent of the carbon source used for growth [40]. The primary candidate clustering with the PDH unit, Cym1p, is annotated in SGD as cytosolic metalloprotease; computational analysis of large-scale protein-protein interaction data suggests a possible role in pyruvate metabolism. Large-scale localization studies, computational prediction of mitochondrial signal peptides and co-purification with mitochondrial proteins in proteomic assays support for Cym1p a mitochondrial localization. The mitochondrial alpha-ketoglutarate dehydrogenase complex (KGD) catalyzes a key step in the tricarboxylic acid (TCA) cycle, the oxidative decarboxylation of alpha-ketoglutarate to form succinyl-CoA.

**Folate and glycine metabolism (c46).** This module includes the glycine cleavage complex (GCV) involved in providing single carbon units for the biosynthesis of purines, thymidylates, serine, methionine, and *N*-formylmethionyl tRNA [41]. The importance of this complex is supported by the large number of human diseases and disorders associated with folate deficiency and disturbances in folate-mediated reactions. The module also includes proteins involved in the synthesis of C1-tetrahydrofolate, serine and glycine.

**TOM complex (c49) and Tim23/Tim22 complex (c16).** The TOM complex contains proteins of the mitochondrial pre-protein translocase machinery across the outer membrane. The TIM complex contains proteins of the two translocases across the inner membrane, Tim22 and Tim23, together with proteins of the mitochondrial intermembrane space protein transporter complex. The TOM and TIM modules are defined mainly by evidence of physical protein-protein interactions. The TOM complex contains import receptors for the initial recognition of preproteins as well as membrane-embedded components that form the general import pore which facilitates the translocation of nuclear-encoded mitochondrial preproteins across the outer membrane [42]. The primary candidate, Sam35p, has strong evidences of mitochondrial localization from large-scale datasets [10,12,14]. Indeed single gene studies had recently shown the Sam35p belongs to the mitochondrial protein sorting and assembly machinery [43]. If the nuclear-encoded mitochondrial preproteins contain a presequence, after crossing the outer membrane, the proteins are transferred to the presequence translocase of the inner membrane, the Tim23 complex. Some inner membrane proteins which only contain internal targeting signals are imported with the assistance of the pore-forming Tim22 complex [44,45]. Primary candidates in the Tim23/Tim22 complex are Tim21p and Pbp1p. Tim21p has strong evidences of mitochondrial localization by GFP-tagged localization, proteomics study on purified mitochondria and computational prediction of a mitochondrial signal peptide. Large-scale localization studies have proposed for Pbp1p different localizations, but it does show a growth defect on respiratory carbon sources. The protein interacts with poly(A)-binding protein Pab1p and it is proposed to be involved in the control of mRNA polyadenylation. Pbp1p was found to rescue the petite-negative phenotype of cells lacking components of the mitochondrial protein import machinery [46]. In general neither ATP exporters nor protein transport systems such as those that recruit proteins into the mitochondria (TOM and TIM) are found among free-living bacteria. This would suggest that the ancestral endosymbiont could not export ATP or import proteins, as do modern mitochondria and these functions probably have descended almost exclusively from eukaryotic genes [47].

**Fusion/fission (c64).** This module includes known mitochondrial proteins involved in mitochondrial morphogenesis. The mitochondrial compartment of budding yeast is a highly dynamic net-like structure of tubules and the maintenance of extended mitochondrial structures in all cell types requires a balance of fission and fusion events [48]. Components of the fusion machinery in this module are Mgm1p, Ugo1p, Fzo1p [49] while components of the fission machinery are Dnm1p, Fis1p and Mdv1p. The module shows a respiratory-specific deletion phenotype on NF carbon sources in accordance with other studies which show that mitochondrial morphology and distribution reflect the energy requirements of the cell [9]. Like their free-living ancestors mitochondria divide. Genes in this module do not show any proteobacterial origin. Indeed mitochondria in fungi, plants, and animals appear to have lost division components of bacterial origin [50].

**Iron-sulfur cluster assembly (c20).** This module includes known mitochondrial components of the Fe/S cluster biosynthesis machinery as well as mitochondrial chaperones. In eukaryotes, mitochondria execute a central task in the assembly of cellular iron-sulfur (Fe/S) proteins. The organelle synthesizes its own set of Fe/S proteins and it initiates the generation of extra-mitochondrial Fe/S proteins [51]. Cytosolic proteins involved in the incorporation of the Fe–S clusters into cytosolic apoproteins have not yet been identified. Such cytosolic components might assist the specific delivery of the labile Fe–S clusters into apoproteins after release from mitochondria, a process that might involve cytosolic chaperones that stabilize the Fe–S apoproteins before the Fe–S clusters are incorporated.

**Arginine biosynthesis (c51)**. In yeast *Saccharomyces cerevisiae* and the other Ascomycetes studied to date, the enzymes involved in the first steps of this pathway are located in mitochondria [52]. The arginine biosynthesis pathway starts in the mitochondria from one L-glutamate molecule to N-acetylglutamate (Arg2p) through a kinase/reductase enzyme (Arg5,6p), an aminotransferase (Arg8p) and acetyltransferase (Ecm40p) till a molecule of ornithine that can be exported to the cytosol (Ort1p). The remaining steps of this pathway are carried out in the cytosol (see the glutamine family metabolism module).

**Nuclear/mitochondria-localized modules**

**Response to DNA damage (c35).** This module is annotated as sharing a nuclear-mitochondrial localization. Three proteins are annotated in the MitoP2 reference set: Rim1p, Mgm101p and Vas1p. Rim1p is a single-stranded DNA-binding protein essential for mitochondrial genome maintenance and involved in mitochondrial DNA replication [53]. Mgm101p is a protein involved in mitochondrial genome maintenance and a component of the mitochondrial nucleoid, required for the repair of oxidative mtDNA damage [54]. Vas1p is annotated in SGD as having a dual mitochondrial-cytoplasmic localization, functionally active as valyl-tRNA synthetase. Vas1p is included in this module by evidences of TAP-tagged co-purification with Rnh1 and other components of this module (BIND database). Rnh1p is the ribonuclease H1 that removes RNA primers during Okazaki fragment synthesis. Rfa2p is a subunit of heterotrimeric replication factor A (RF-A), which is a highly conserved single-stranded DNA binding protein involved in DNA replication, repair, and recombination. Lcd1p is an essential protein required for the DNA integrity checkpoint pathway and interacts physically with Mec1p [55]. Mec1 is a primary candidate according to proteomic study and orthology to *Rickettsia prowazekii.* Although the two classes of proteins, reference set and secondary candidate, have a different annotation regarding their localization, experimental evidence of co-purification to the Rnh1p TAP-tagged protein might suggest alternative roles for these proteins.

**Nuclear localized modules**

**DNA replication (c41).** This module is annotated as nuclear localizing and includes the ribonucleotide-diphosphate reductase (RNR) complex. The RNR complex catalyzes the rate-limiting step in dNTP synthesis and is regulated by DNA replication and DNA damage checkpoint pathways [56]. Two proteins, Rnr3p and Rnr4p, are primary candidates: they show bacterial orthology to *Rickettsia prowazekii* and protein-protein interaction to known mitochondrial proteins [57]. Rnr3p is differentially expressed [1] and copurifies with mitochondria [12]. Rnr4p has a mutant phenotype [8,9]. All of these evidences are in line with recent findings that components of the RNR complex can influence replication, repair, or stability of mtDNA through the modulation of dNTP pools [58]. Mutations in RNR genes result in increased petite mutant formation; overexpression of RNR1 rescues the petite-induction phenotype of a point mutation in mtDNA polymerase g, indicating that the mitochondrial replication or repair machinery is indeed responsive to alterations in dNTP pool via RNR activity. This module also contains few known mitochondrial localizing proteins: Mam33p, Adk1p and Adk2p. Mam33p encodes an acidic protein of unknown function. The inclusion of Mam33p in this module is supported by evidences of physical interaction [15,59]. Adk1p is a protein with a known dual cytoplasmic-mitochondrial localization with adenylate kinase activity required for purine metabolism. Adk2p is a mitochondrial adenylate kinase that may serve as a back-up for synthesizing GTP or ADP depending on metabolic conditions.

**Mismatch repair (c105).** The Yeast genome encodes four mismatch repair MutL proteins: Pms1p, Mlh1p, Mlh2p and Mlh3p. These proteins form heterocomplexes: Mlh1p-Mlh2p, Mlh1p-Mlh3p and Mlh1p-Pms1p. They play an important role during meiotic recombination [60-62]. Mlh1p was found to co-purify with mitochondrial proteins in a proteomic study by Sickmann et al. and the deletion mutant shows growth defect on respiratory carbon sources. As already shown, proteins involved in repair of oxidative DNA damage often show a dual mitochondrial-nuclear localization but up to date there is no published evidence that Mlh1p is imported into the organelle. The module includes a mitochondrial protein, Msh1p, involved in the repair of mitochondrial DNA that is connected by evidences of co-occurrence in PubMed abstracts with nuclear components of the mismatch repair pathway. The mismatch repair system is highly conserved from bacteria to humans. Mutations in *MLH1*, *MLH3* and *PMS1* have been linked to hereditary non-polyposis colorectal cancer (HNPCC) [63,64].

**Cytoplasm/mitochondria-localized modules**

**Fatty acid synthesis (c12).** The module includes proteins involved in fatty acid biosynthesis. Yeast contains two distinct fatty-acid synthases (FAS) acting specifically in the cytoplasm (type I FAS) and in the mitochondrial matrix (type II FAS). Cytoplasmic FAS is an essential multifunctional enzyme composed of a beta (Fas1p) and an alpha subunit (Fas2p) that synthesizes most of cellular fatty acids. Both enzymes are primary candidates in this module according to localization evidence from proteomic assays and orthology to *Rickettsia prowazekii*. A specific role has been assigned to the mitochondrial FAS in the synthesis of lipoic acid. Lipoic acid is a universal cofactor critical for the function of mitochondrial multi-enzyme complexes, including pyruvate dehydrogenase (PDH), α-ketoglutarate dehydrogenase (KGDH) and glycine decarboxylase [65]. Cem1p is a component of the mitochondrial type II FAS [66]. Hfa1p is the third primary candidate. Hfa1p has been recently included in the MitoP2 reference set as a result of a study that confirmed the mitochondrial localization of Hfa1p and its function as an organelle-specific acetyl-CoA carboxylase [67]. Mutations in HFA1 abolish mitochondrial fatty acid synthesis, deletion mutants exhibit a growth defect on non-fermentable carbon sources, Hfa1p has been identified by proteomics and tag-localized to mitochondria.

**Glycerol metabolism (c48).** This module includes cytoplasmic and mitochondrial enzymes involved in glycerol metabolism. Cytosolic glycerol-3-phosphate is directly oxidized by a mitochondrial inner membrane bound glycerol-3-phosphate-ubiquinone oxidoreductase, Gut2p, which in association with cytosolic enzymes, Gpd1p and Gpd2p, creates a glycerol-3-phosphate shuttle involved in redox adjustment [33]. The expression of GPD1 is regulated during respiratory metabolism, as reported by Prokisch et al. [1] and Derisi et al. [20]. Gpd2p is also a primary candidate identified by proteomic studies on purified mitochondria, prediction of a signal peptide and large-scale subcellular localization to mitochondria [11]. The cytoplasmic enzyme Gut1p converts glycerol to glycerol-3-phosphate; its expression is repressed in the presence of glucose and its derepression happens on non-fermentable carbon sources as evidenced by a single gene study [68] and large scale expression profiles [1,9]. In addition, prediction of a signal peptide and GFP-tagged protein localization suggest for this enzyme a mitochondrial localization. In this module, three known mitochondrial enzymes (Crd1p, Cds1p, Pgs1p) are also involved in the synthesis of phospholipids, a pathway that starts from glycerol 3-phosphate. All three are involved in the synthesis of cardiolipin, an important constituent of mitochondrial membranes.

**Alcohol/aldehyde dehydrogenase (c103).** This module includes three alcohol dehydrogenases that reduce acetaldehyde to ethanol. *S. cerevisiae* has at least two cytosolic isoenzymes of alcohol dehydrogenase and one mitochondrial isoenzyme. Adh1p is primarily involved in alcoholic fermentation required for the reduction of acetaldehyde to ethanol. Adh3p is a mitochondrial alcohol dehydrogenase (isozyme III) involved in the shuttling of mitochondrial NADH to the cytosol under anaerobic conditions and ethanol production [69]. Adh4p is an alcohol dehydrogenase type IV for which a mitochondrial localization can be suggested due to a large-scale localization study, proteomic assay and prediction of a mitochondrial signal peptide. Ach1p is an acetyl-coA hydrolase, primarily localized to mitochondria; required for acetate utilization. Sfa1p is a bifunctional enzyme containing both alcohol dehydrogenase and glutathione-dependent formaldehyde dehydrogenase activities; it functions in formaldehyde detoxification and formation of long chain and complex alcohols and was identified in the proteomic study on purified mitochondria. Ald6p is a mitochondrial-cytosolic [70] aldehyde dehydrogenase that converts acetaldehyde to acetate and it is constitutively expressed. Two other aldehyde dehydrogenases function in the mitochondria. Ald5p is involved in regulation or biosynthesis of electron transport chain components and acetate formation and is constitutively expressed. Ald4p is required for growth on ethanol to convert acetaldehyde to acetate, expression is glucose repressed and is up-regulated when grown on ethanol. Two cytosolic acetyl-CoA synthetases, Acs1p and Acs2p catalyze the formation of acetyl-CoA from acetate. *ACS1* mRNA levels are reduced when cells are grown on glucose and are increased when grown on NF carbon sources. Large scale localization study and proteomic assay suggest a mitochondrial localization. Acs2p is required for growth on glucose and expressed under anaerobic conditions. The gene shows orthology to known mitochondrial protein in *Neurospora* and *Rickettsia*.

**tRNA aminoacylation (c80).** The tRNA aminoacylation module includes four known mitochondrial localizing proteins with aminoacyl-tRNA ligase activity (Aat1p, Msy1p, Msf1p, Msd1p) and the essential cytoplasmic phenylalanyl-tRNA synthetase complex (Fsr1p, Fsr2p). The module is defined by functional associations derived from import of association links by KEGG database. All null mutants in the mitochondrial localized proteins show a severe phenotype when grown on NF conditions. The primary candidate protein, Aat2p, encodes an [aspartate aminotransferase](http://db.yeastgenome.org/cgi-bin/GO/go.pl?goid=0004069) that catalyzes the reversible transfer of the amino group from L-aspartate to 2-oxoglutarate to form oxaloacetate and L-glutamate. Aat2p is usually found in the cytoplasm, but is targeted to peroxisome in cells grown in oleate. It also shares sequence similarity with the yeast mitochondrial aspartate aminotransferase, Aat1p. Yeast strains lacking AAT2 are viable in rich media, but require aspartic acid in minimal media and they exhibit growth defect on respiratory carbon sources. Both Aat1p and Aat2p show proteobacterial origin and the orthologous genes in human and *Neurospora crassa* have a mitochondrial localization.

**Cytoplasm-localized modules**

**Cell wall organization and biogenesis (c54)**. This module includes proteins involved in cell wall synthesis and maintenance such as Gsc2p, Fks1p and Fen1p. Gsc2p is a catalytic subunit of 1,3-beta-glucan synthase that has similarity to an alternate catalytic subunit, Fks1p. Fks1p is the catalytic subunit of 1,3-beta-D-glucan synthase and Fen1p is a fatty acid elongase, involved in sphingolipid biosynthesis; mutations in Fen1p have regulatory effects on 1,3-beta-glucan synthase [71]. Three primary candidates, including Fks1p, Phr1p and Gas1p show evidence of a mitochondrial localization. Among the primary candidates Gas1p is particularly interesting because its molecular function and localization in cell wall assembly is well known, but it might also have a mitochondrial localization based on our data integration. Gas1p is a beta-1,3-glucanosyltransferase, required for cell wall assembly. Evidence from single gene study has localized the enzyme to the cell surface via a glycosylphosphatidylinositol (GPI) anchor [72]. According to proteomic studies on the purified mitochondrial organelle and a large scale GFP-tagged protein localization study, a dual mitochondrial localization might be suggested for Gas1p. Genome-wide analysis of mRNAs targeted to yeast mitochondria also found the mRNA for Gas1p localized to mitochondrion-bound polysomes. The inclusion of Phr1p, a DNA photolyase induced by DNA damage [73], is due to a text-mining artifact.

**Vacuolar acidification (c58).** This module includes the vacuolar H (+)-ATPase complex (V-ATPase) and two subunit of the RAVE complex (Rav1p, Rav2p). The V-ATPase consists of two separable domains. The V_1_ domain has eight known subunits, is peripherally associated with the vacuolar membrane, and catalyzes ATP hydrolysis. The V_0_ domain is an integral membrane structure of five subunits and transports protons across the membrane. Association of the two complexes is essential for ATP-driven proton transport and is regulated post-translationally in response to glucose concentration. The RAVE complex associates with the V1 domain and promotes assembly and reassembly of the V-ATPase in a glucose-dependent manner [74]. V-ATPases have been identified in many eukaryotes, where they acidify intracellular vacuolar compartments [75]. Vacuolar acidification is important for many cellular processes, including endocytosis and targeting of newly synthesized lysosomal enzymes. Other lines of evidence report that the vacuolar H(+)-ATPase of Saccharomyces cerevisiae is required for efficient copper detoxification, respiration, and iron metabolism [76,77]. The function of yeast vacuoles in iron and copper metabolism could explain vacuolar mutants having a petite phenotype and impaired respiration [77-79]. Some of the components of the V_1_ and V_0_ domains are primary candidates in our parts list. Rather than a prediction of mitochondrial localization, data from large scale studies suggest for these primary candidates an involvement in mitochondrial function. Null mutants exhibit growth defect on respiratory carbon sources and petite phenotypes [8,9].

**Cytosolic ribosome (c132).** This module includes 13 structural constituents of the small (40S) and large (60S) ribosomal subunits. All of these proteins are primary candidates in our parts list: they were co-purified with mitochondria (Prokisch and Sickmann) and found interacting with mitochondrial ribosomal proteins. This observation could hint for a dual localization of some ribosomal proteins. In addition, these proteins might play a role in mitochondria-ribosome interactions[80,81] Experiments conducted already from 1972 to 1975 observed that there are at least two classes of ribosome binding sites of different affinity for mitochondria. The cytoplasmic ribosome might be able to distinguish between different mRNA species to direct the mitochondrial import of proteins. Moreover the candidate proteins in this module might be specifically involved in the cotranslational import of proteins from mitochondrion-bound polysomes [21]. Contrary to modules with a NF-impaired deletion phenotypes, null mutants in components of the cytosolic ribosome module show increased survival on NF media compared to standard YPD media, consistent with down-regulation of genes involved in protein synthesis during respiration [1,20].

**Glutamine family metabolism (c24).** This module includes enzymes involved in the cytosolic step of the arginine biosynthethic pathway (Cpa1p and Cpa2p, components of the carbamoyl-phosphate synthase complex) and the superpathway of histidine, purine, and pyrimidine biosynthesis (Ura2p and Ura7p). Both these pathways start from L-glutamate or L-glutamine molecules as substrates. Cpa1p and Cpa2p are primary candidates based on orthology to *Neurospora* and human genes with mitochondrial localization. The carbamoyl phosphate is required to make citrulline, the precursor of ornithine. Up to date it is known that in *S. cerevisiae*, ornithine is exported to the cytosol for further processing to arginine, whereas in a number of other yeasts this happens in the mitochondria although the meaning of this difference in localization is not obvious [82]. A similar case is the Ura2p enzyme. Ura2p is a primary candidate for mitochondrial localization since it was identified by two proteomic studies performed on purified mitochondria but alternative approaches based on the detection of the protein itself localized the bulk of Ura2 to the cytoplasm (in this study the signals associated with the nucleus, mitochondria or vacuoles were close to or at the background level) [82]. URA2 is found to be down-regulated in NF growth conditions. As expected this module is connected at high confidence to the mitochondrial module involved in arginine synthesis and the mitochondrial-cytoplasmic module involved in glutamate synthesis. The inclusion of Ctp1p, a mitochondrial inner membrane citrate transporter, is due to a text-mining artifact.

**References**

1. Prokisch H, Scharfe C, Camp DG, 2nd, Xiao W, David L, et al. (2004) Integrative analysis of the mitochondrial proteome in yeast. PLoS Biol 2: e160.

2. Prokisch H, Andreoli C, Ahting U, Heiss K, Ruepp A, et al. (2006) MitoP2: the mitochondrial proteome database--now including mouse data. Nucleic Acids Res 34: D705-711.

3. von Mering C, Krause R, Snel B, Cornell M, Oliver SG, et al. (2002) Comparative assessment of large-scale data sets of protein-protein interactions. Nature 417: 399-403.

4. de Lichtenberg U, Jensen LJ, Brunak S, Bork P (2005) Dynamic complex formation during the yeast cell cycle. Science 307: 724-727.

5. Jensen LJ, Lagarde J, von Mering C, Bork P (2004) ArrayProspector: a web resource of functional associations inferred from microarray expression data. Nucleic Acids Res 32: W445-448.

6. Krogan NJ, Cagney G, Yu H, Zhong G, Guo X, et al. (2006) Global landscape of protein complexes in the yeast Saccharomyces cerevisiae. Nature 440: 637-643.

7. Gavin AC, Aloy P, Grandi P, Krause R, Boesche M, et al. (2006) Proteome survey reveals modularity of the yeast cell machinery. Nature 440: 631-636.

8. Steinmetz LM, Scharfe C, Deutschbauer AM, Mokranjac D, Herman ZS, et al. (2002) Systematic screen for human disease genes in yeast. Nat Genet 31: 400-404.

9. Dimmer KS, Fritz S, Fuchs F, Messerschmitt M, Weinbach N, et al. (2002) Genetic basis of mitochondrial function and morphology in *Saccharomyces cerevisiae*. Mol Biol Cell 13: 847-853.

10. Huh WK, Falvo JV, Gerke LC, Carroll AS, Howson RW, et al. (2003) Global analysis of protein localization in budding yeast. Nature 425: 686-691.

11. Kumar A, Agarwal S, Heyman JA, Matson S, Heidtman M, et al. (2002) Subcellular localization of the yeast proteome. Genes Dev 16: 707-719.

12. Sickmann A, Reinders J, Wagner Y, Joppich C, Zahedi R, et al. (2003) The proteome of *Saccharomyces cerevisiae* mitochondria. Proc Natl Acad Sci U S A 100: 13207-13212.

13. Ohlmeier S, Kastaniotis AJ, Hiltunen JK, Bergmann U (2003) The yeast mitochondrial proteome - a study of fermentative and respiratory growth. J Biol Chem.

14. Pflieger D, Le Caer JP, Lemaire C, Bernard BA, Dujardin G, et al. (2002) Systematic identification of mitochondrial proteins by LC-MS/MS. Anal Chem 74: 2400-2406.

15. Gavin AC, Bosche M, Krause R, Grandi P, Marzioch M, et al. (2002) Functional organization of the yeast proteome by systematic analysis of protein complexes. Nature 415: 141-147.

16. Ho Y, Gruhler A, Heilbut A, Bader GD, Moore L, et al. (2002) Systematic identification of protein complexes in *Saccharomyces cerevisiae* by mass spectrometry. Nature 415: 180-183.

17. Ito T, Chiba T, Ozawa R, Yoshida M, Hattori M, et al. (2001) A comprehensive two-hybrid analysis to explore the yeast protein interactome. Proc Natl Acad Sci U S A 98: 4569-4574.

18. Uetz P, Giot L, Cagney G, Mansfield TA, Judson RS, et al. (2000) A comprehensive analysis of protein-protein interactions in *Saccharomyces cerevisiae*. Nature 403: 623-627.

19. Lascaris R, Bussemaker HJ, Boorsma A, Piper M, van der Spek H, et al. (2003) Hap4p overexpression in glucose-grown *Saccharomyces cerevisiae* induces cells to enter a novel metabolic state. Genome Biol 4: R3.

20. DeRisi JL, Iyer VR, Brown PO (1997) Exploring the metabolic and genetic control of gene expression on a genomic scale. Science 278: 680-686.

21. Marc P, Margeot A, Devaux F, Blugeon C, Corral-Debrinski M, et al. (2002) Genome-wide analysis of mRNAs targeted to yeast mitochondria. EMBO Rep 3: 159-164.

22. Small I, Peeters N, Legeai F, Lurin C (2004) Predotar: A tool for rapidly screening proteomes for N-terminal targeting sequences. Proteomics 4: 1581-1590.

23. Claros MG, Vincens P (1996) Computational method to predict mitochondrially imported proteins and their targeting sequences. European Journal of Biochemistry 241: 779-786.

24. Guda C, Fahy E, Subramaniam S (2004) MITOPRED: a genome-scale method for prediction of nucleus-encoded mitochondrial proteins. Bioinformatics 20: 1785-1794.

25. Drawid A, Gerstein M (2000) A Bayesian system integrating expression data with sequence patterns for localizing proteins: comprehensive application to the yeast genome. J Mol Biol 301: 1059-1075.

26. Nakai K, Horton P (1999) PSORT: a program for detecting sorting signals in proteins and predicting their subcellular localization. Trends Biochem Sci 24: 34-36.

27. Andreoli C, Prokisch H, Hortnagel K, Mueller JC, Munsterkotter M, et al. (2004) MitoP2, an integrated database on mitochondrial proteins in yeast and man. Nucleic Acids Res 32: D459-462.

28. Andersson SG, Zomorodipour A, Andersson JO, Sicheritz-Ponten T, Alsmark UC, et al. (1998) The genome sequence of *Rickettsia prowazekii* and the origin of mitochondria. Nature 396: 133-140.

29. Katinka MD, Duprat S, Cornillot E, Metenier G, Thomarat F, et al. (2001) Genome sequence and gene compaction of the eukaryote parasite *Encephalitozoon cuniculi*. Nature 414: 450-453.

30. Salinas K, Wierzbicki S, Zhou L, Schmitt ME (2005) Characterization and purification of *Saccharomyces cerevisiae* RNase MRP reveals a new unique protein component. J Biol Chem 280: 11352-11360.

31. Chacinska A, Boguta M (2000) Coupling of mitochondrial translation with the formation of respiratory complexes in yeast mitochondria. Acta Biochim Pol 47: 973-991.

32. Sato H, Miyakawa I (2004) A 22 kDa protein specific for yeast mitochondrial nucleoids is an unidentified putative ribosomal protein encoded in open reading frame YGL068W. Protoplasma 223: 175-182.

33. Grandier-Vazeille X, Bathany K, Chaignepain S, Camougrand N, Manon S, et al. (2001) Yeast mitochondrial dehydrogenases are associated in a supramolecular complex. Biochemistry 40: 9758-9769.

34. Liu Z, Butow RA (1999) A transcriptional switch in the expression of yeast tricarboxylic acid cycle genes in response to a reduction or loss of respiratory function. Mol Cell Biol 19: 6720-6728.

35. Cassanova N, O'Brien KM, Stahl BT, McClure T, Poyton RO (2005) Yeast flavohemoglobin, a nitric oxide oxidoreductase, is located in both the cytosol and the mitochondrial matrix: effects of respiration, anoxia, and the mitochondrial genome on its intracellular level and distribution. J Biol Chem 280: 7645-7653.

36. Hodge MR, Kim G, Singh K, Cumsky MG (1989) Inverse regulation of the yeast COX5 genes by oxygen and heme. Mol Cell Biol 9: 1958-1964.

37. Pickova A, Potocky M, Houstek J (2005) Assembly factors of F1FO-ATP synthase across genomes. Proteins 59: 393-402.

38. Devenish RJ, Prescott M, Roucou X, Nagley P (2000) Insights into ATP synthase assembly and function through the molecular genetic manipulation of subunits of the yeast mitochondrial enzyme complex. Biochim Biophys Acta 1458: 428-442.

39. Pronk JT, Yde Steensma H, Van Dijken JP (1996) Pyruvate metabolism in Saccharomyces cerevisiae. Yeast 12: 1607-1633.

40. Wenzel TJ, Luttik MA, van den Berg JA, de Steensma HY (1993) Regulation of the PDA1 gene encoding the E1 alpha subunit of the pyruvate dehydrogenase complex from Saccharomyces cerevisiae. Eur J Biochem 218: 405-411.

41. Gelling CL, Piper MD, Hong SP, Kornfeld GD, Dawes IW (2004) Identification of a novel one-carbon metabolism regulon in Saccharomyces cerevisiae. J Biol Chem 279: 7072-7081.

42. Stan T, Ahting U, Dembowski M, Kunkele KP, Nussberger S, et al. (2000) Recognition of preproteins by the isolated TOM complex of mitochondria. Embo J 19: 4895-4902.

43. Milenkovic D, Kozjak V, Wiedemann N, Lohaus C, Meyer HE, et al. (2004) Sam35 of the mitochondrial protein sorting and assembly machinery is a peripheral outer membrane protein essential for cell viability. J Biol Chem 279: 22781-22785.

44. Truscott KN, Brandner K, Pfanner N (2003) Mechanisms of protein import into mitochondria. Curr Biol 13: R326-337.

45. Taylor RD, Pfanner N (2004) The protein import and assembly machinery of the mitochondrial outer membrane. Biochim Biophys Acta 1658: 37-43.

46. Dunn CD, Jensen RE (2003) Suppression of a defect in mitochondrial protein import identifies cytosolic proteins required for viability of yeast cells lacking mitochondrial DNA. Genetics 165: 35-45.

47. Kurland CG, Andersson SG (2000) Origin and evolution of the mitochondrial proteome. Microbiol Mol Biol Rev 64: 786-820.

48. Scott SV, Cassidy-Stone A, Meeusen SL, Nunnari J (2003) Staying in aerobic shape: how the structural integrity of mitochondria and mitochondrial DNA is maintained. Curr Opin Cell Biol 15: 482-488.

49. Sesaki H, Jensen RE (2004) Ugo1p links the Fzo1p and Mgm1p GTPases for mitochondrial fusion. J Biol Chem 279: 28298-28303.

50. Osteryoung KW, Nunnari J (2003) The division of endosymbiotic organelles. Science 302: 1698-1704.

51. Lill R, Kispal G (2000) Maturation of cellular Fe-S proteins: an essential function of mitochondria. Trends Biochem Sci 25: 352-356.

52. Abadjieva A, Pauwels K, Hilven P, Crabeel M (2001) A new yeast metabolon involving at least the two first enzymes of arginine biosynthesis: acetylglutamate synthase activity requires complex formation with acetylglutamate kinase. J Biol Chem 276: 42869-42880.

53. Van Dyck E, Foury F, Stillman B, Brill SJ (1992) A single-stranded DNA binding protein required for mitochondrial DNA replication in S. cerevisiae is homologous to E. coli SSB. Embo J 11: 3421-3430.

54. Meeusen S, Tieu Q, Wong E, Weiss E, Schieltz D, et al. (1999) Mgm101p is a novel component of the mitochondrial nucleoid that binds DNA and is required for the repair of oxidatively damaged mitochondrial DNA. J Cell Biol 145: 291-304.

55. Wakayama T, Kondo T, Ando S, Matsumoto K, Sugimoto K (2001) Pie1, a protein interacting with Mec1, controls cell growth and checkpoint responses in Saccharomyces cerevisiae. Mol Cell Biol 21: 755-764.

56. Yao R, Zhang Z, An X, Bucci B, Perlstein DL, et al. (2003) Subcellular localization of yeast ribonucleotide reductase regulated by the DNA replication and damage checkpoint pathways. Proc Natl Acad Sci U S A 100: 6628-6633.

57. van den Ent F, Amos LA, Lowe J (2001) Prokaryotic origin of the actin cytoskeleton. Nature 413: 39-44.

58. O'Rourke TW, Doudican NA, Zhang H, Eaton JS, Doetsch PW, et al. (2005) Differential involvement of the related DNA helicases Pif1p and Rrm3p in mtDNA point mutagenesis and stability. Gene 354: 86-92.

59. Puig O, Caspary F, Rigaut G, Rutz B, Bouveret E, et al. (2001) The tandem affinity purification (TAP) method: a general procedure of protein complex purification. Methods 24: 218-229.

60. Earley MC, Crouse GF (1998) The role of mismatch repair in the prevention of base pair mutations in Saccharomyces cerevisiae. Proc Natl Acad Sci U S A 95: 15487-15491.

61. Hall MC, Shcherbakova PV, Fortune JM, Borchers CH, Dial JM, et al. (2003) DNA binding by yeast Mlh1 and Pms1: implications for DNA mismatch repair. Nucleic Acids Res 31: 2025-2034.

62. Kang D, Hamasaki N (2002) Maintenance of mitochondrial DNA integrity: repair and degradation. Curr Genet 41: 311-322.

63. Muller A, Fishel R (2002) Mismatch repair and the hereditary non-polyposis colorectal cancer syndrome (HNPCC). Cancer Invest 20: 102-109.

64. Heinen CD, Schmutte C, Fishel R (2002) DNA repair and tumorigenesis: lessons from hereditary cancer syndromes. Cancer Biol Ther 1: 477-485.

65. Hiltunen JK, Okubo F, Kursu VA, Autio KJ, Kastaniotis AJ (2005) Mitochondrial fatty acid synthesis and maintenance of respiratory competent mitochondria in yeast. Biochem Soc Trans 33: 1162-1165.

66. Harington A, Herbert CJ, Tung B, Getz GS, Slonimski PP (1993) Identification of a new nuclear gene (CEM1) encoding a protein homologous to a beta-keto-acyl synthase which is essential for mitochondrial respiration in Saccharomyces cerevisiae. Mol Microbiol 9: 545-555.

67. Hoja U, Marthol S, Hofmann J, Stegner S, Schulz R, et al. (2004) HFA1 encoding an organelle-specific acetyl-CoA carboxylase controls mitochondrial fatty acid synthesis in Saccharomyces cerevisiae. J Biol Chem 279: 21779-21786.

68. Grauslund M, Lopes JM, Ronnow B (1999) Expression of GUT1, which encodes glycerol kinase in Saccharomyces cerevisiae, is controlled by the positive regulators Adr1p, Ino2p and Ino4p and the negative regulator Opi1p in a carbon source-dependent fashion. Nucleic Acids Res 27: 4391-4398.

69. Bakker BM, Bro C, Kotter P, Luttik MA, van Dijken JP, et al. (2000) The mitochondrial alcohol dehydrogenase Adh3p is involved in a redox shuttle in Saccharomyces cerevisiae. J Bacteriol 182: 4730-4737.

70. Saigal D, Cunningham SJ, Farres J, Weiner H (1991) Molecular cloning of the mitochondrial aldehyde dehydrogenase gene of Saccharomyces cerevisiae by genetic complementation. J Bacteriol 173: 3199-3208.

71. Abe M, Nishida I, Minemura M, Qadota H, Seyama Y, et al. (2001) Yeast 1,3-beta-glucan synthase activity is inhibited by phytosphingosine localized to the endoplasmic reticulum. J Biol Chem 276: 26923-26930.

72. Nuoffer C, Jeno P, Conzelmann A, Riezman H (1991) Determinants for glycophospholipid anchoring of the Saccharomyces cerevisiae GAS1 protein to the plasma membrane. Mol Cell Biol 11: 27-37.

73. Sebastian J, Sancar GB (1991) A damage-responsive DNA binding protein regulates transcription of the yeast DNA repair gene PHR1. Proc Natl Acad Sci U S A 88: 11251-11255.

74. Seol JH, Shevchenko A, Deshaies RJ (2001) Skp1 forms multiple protein complexes, including RAVE, a regulator of V-ATPase assembly. Nat Cell Biol 3: 384-391.

75. Forgac M (1999) Structure and properties of the vacuolar (H+)-ATPases. J Biol Chem 274: 12951-12954.

76. Nelson N (2003) A journey from mammals to yeast with vacuolar H+-ATPase (V-ATPase). J Bioenerg Biomembr 35: 281-289.

77. Eide DJ, Bridgham JT, Zhao Z, Mattoon JR (1993) The vacuolar H(+)-ATPase of Saccharomyces cerevisiae is required for efficient copper detoxification, mitochondrial function, and iron metabolism. Mol Gen Genet 241: 447-456.

78. Chen OS, Hemenway S, Kaplan J (2002) Genetic analysis of iron citrate toxicity in yeast: implications for mammalian iron homeostasis. Proc Natl Acad Sci U S A 99: 16922-16927.

79. Szczypka MS, Zhu Z, Silar P, Thiele DJ (1997) *Saccharomyces cerevisiae* mutants altered in vacuole function are defective in copper detoxification and iron-responsive gene transcription. Yeast 13: 1423-1435.

80. Kellems RE, Butow RA (1972) Cytoplasmic-type 80 S ribosomes associated with yeast mitochondria. I. Evidence for ribosome binding sites on yeast mitochondria. J Biol Chem 247: 8043-8050.

81. Margeot A, Garcia M, Wang W, Tetaud E, di Rago JP, et al. (2005) Why are many mRNAs translated to the vicinity of mitochondria: a role in protein complex assembly? Gene 354: 64-71.

82. Jauniaux JC, Urrestarazu LA, Wiame JM (1978) Arginine metabolism in Saccharomyces cerevisiae: subcellular localization of the enzymes. J Bacteriol 133: 1096-1107.
